# Comprehensive Mechanistic View of the Hydrolysis of Oxadiazole-Based Inhibitors by Histone Deacetylase 6 (HDAC6)

**DOI:** 10.1021/acschembio.3c00212

**Published:** 2023-07-03

**Authors:** Lucia Motlová, Ivan Šnajdr, Zsófia Kutil, Erik Andris, Jakub Ptáček, Adéla Novotná, Zora Nováková, Barbora Havlínová, Werner Tueckmantel, Helena Dráberová, Pavel Majer, Mike Schutkowski, Alan Kozikowski, Lubomír Rulíšek, Cyril Bařinka

**Affiliations:** † Institute of Biotechnology of the Czech Academy of Sciences, BIOCEV, Prumyslova 595, 252 50 Vestec, Czech Republic; ‡ Institute of Organic Chemistry and Biochemistry of the Czech Academy of Sciences, Flemingovo náměstí 2, 166 10 Prague 6, Czech Republic; § StarWise Therapeutics LLC, University Research Park, Inc., Madison, Wisconsin 53719, United States; ∥ Department of Enzymology, Charles Tanford Protein Center, Institute of Biochemistry and Biotechnology, Martin-Luther-University Halle-Wittenberg, 06120 Halle, Germany

## Abstract

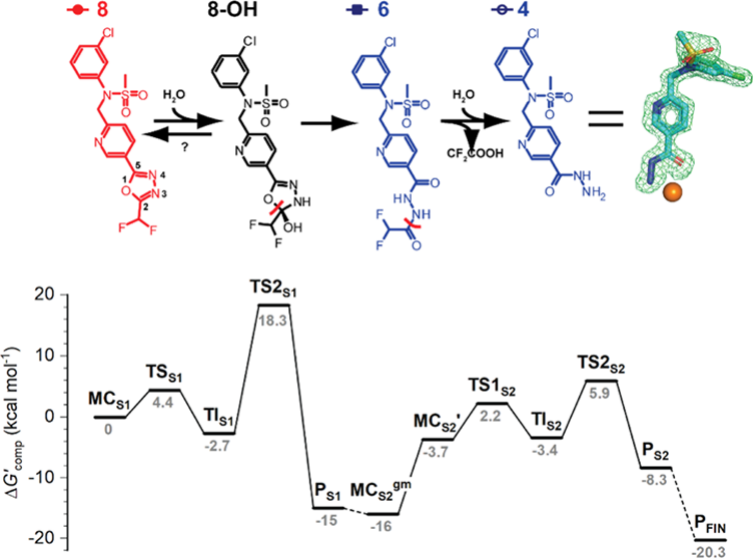

Histone deacetylase (HDAC) inhibitors used in the clinic typically contain a hydroxamate zinc-binding group (ZBG). However, more recent work has shown that the use of alternative ZBGs, and, in particular, the heterocyclic oxadiazoles, can confer higher isoenzyme selectivity and more favorable ADMET profiles. Herein, we report on the synthesis and biochemical, crystallographic, and computational characterization of a series of oxadiazole-based inhibitors selectively targeting the HDAC6 isoform. Surprisingly, but in line with a very recent finding reported in the literature, a crystal structure of the HDAC6/inhibitor complex revealed that hydrolysis of the oxadiazole ring transforms the parent oxadiazole into an acylhydrazide through a sequence of two hydrolytic steps. An identical cleavage pattern was also observed both *in vitro* using the purified HDAC6 enzyme as well as in cellular systems. By employing advanced quantum and molecular mechanics (QM/MM) and QM calculations, we elucidated the mechanistic details of the two hydrolytic steps to obtain a comprehensive mechanistic view of the double hydrolysis of the oxadiazole ring. This was achieved by fully characterizing the reaction coordinate, including identification of the structures of all intermediates and transition states, together with calculations of their respective activation (free) energies. In addition, we ruled out several (intuitively) competing pathways. The computed data (Δ*G*^‡^ ≈ 21 kcal·mol^–1^ for the rate-determining step of the overall dual hydrolysis) are in very good agreement with the experimentally determined rate constants, which *a posteriori* supports the proposed reaction mechanism. We also clearly (and quantitatively) explain the role of the −CF_3_ or −CHF_2_ substituent on the oxadiazole ring, which is a prerequisite for hydrolysis to occur. Overall, our data provide compelling evidence that the oxadiazole warheads can be efficiently transformed within the active sites of target metallohydrolases to afford reaction products possessing distinct selectivity and inhibition profiles.

## Introduction

Zinc-dependent histone deacetylases (HDACs) play critical roles in numerous (patho)physiological processes through the deacylation of lysine residues of their substrate proteins. Since HDACs represent attractive therapeutic targets, the development of HDAC inhibitors (HDACis) has garnered considerable interest in both academia and the pharmaceutical industry. Numerous HDACis have been synthesized or isolated as natural products, and five compounds have been approved as anti-cancer drugs.^1,2^ While pan-specific HDACis are suitable for oncology, isoform-specific compounds, most notably HDAC6-selective inhibitors, are being developed for the long-term treatment of chronic diseases, including neurodegenerative and psychiatric conditions, due to their perceived limited cytotoxicity.^3,4^

Selectivity against the individual HDAC isoforms is achieved *via* the productive combination of a zinc-binding group (ZBG), a linker, and a capping group. Currently, the majority of zinc-dependent HDAC inhibitors, including the approved drugs, contain a hydroxamate function as the zinc-binding group. While contributing to high inhibitory potency, the hydroxamates feature several liabilities, including poor/modest oral bioavailability, low metabolic stability, off-target effects, and long-term toxicity issues.^5−8^ Furthermore, due to the high polarity of the hydroxamate group, the discovery of brain-penetrant HDAC6is has remained challenging. Thus, it was deemed of value to investigate the selectivity of HDAC6is that possess alternative ZBGs. A variety of different ZBGs have been investigated as hydroxamate substitutes, including mercaptoamides, thiols, hydrazides, and substituted oxadiazoles.^9−12^ 1,3,4-Oxadiazole and 1,2,4-oxadiazole moieties as zinc-binding groups have been successfully utilized in the discovery of novel inhibitors of HDACs.^3,13−15^ Notably, some oxadiazole-based HDAC6is developed by the Chong Kun Dang pharmaceutical company have been investigated in axonal transport *in vitro*, which suggests that they may be effective in models of neurodegenerative diseases.^3^ Due to the perceived metabolic stability of the oxadiazoles, the replacement of hydroxamate with an oxadiazole-based ZBG provides an opportunity to refine the ADMET profiles of HDAC6is, especially in regard to their possible genotoxicity, a consideration that owes to the possibility that the hydroxamates may undergo the Lossen rearrangement to generate electrophilic isocyanates.

Structurally, HDAC6 is a zinc-dependent metallohydrolase with an atypical complex domain organization that interacts predominantly with cytosolic substrates, including tubulin, cortactin, and peroxiredoxins.^16−19^ The catalytic activity of HDAC6 toward acetylated substrates has been studied both experimentally and computationally.^16,17,20^ The acetyllysine amide bond of the Michaelis enzyme/substrate complex is first polarized *via* interactions with the active-site zinc ion, then an activated water molecule attacks the carbonyl carbon of the scissile bond, and the resulting tetrahedral intermediate is stabilized *via* interactions with the Y745 hydroxyl group. Finally, the C–N bond in the tetrahedral intermediate is cleaved by the final proton transfer from H574 to the peptide bond amide, and the reaction products are released from the active site of the enzyme. Crystallographic studies have captured snapshots of the aforementioned catalytic cycle, and site-directed mutagenesis has confirmed the critical involvement of Y745 and H574 in intermediate stabilization and proton transfer, respectively.^16,17^ Furthermore, mechanistic details of the single hydrolysis of acylated lysine by HDAC6 have been elucidated by QM and quantum and molecular mechanics (QM/MM) methods that represent unique tools to study the reaction mechanism of metalloproteins.^20^

Still, the question of why and how the enzyme that has evolved to catalyze lysine deacetylation transforms the oxadiazole ring of the inhibitor into an acylhydrazide in a series of two hydrolytic steps has remained elusive to experimental and computational investigations. Clear experimental evidence is provided here by employing X-ray crystallography to observe the product (acylhydrazide) bound in the active site. Moreover, the hydrolysis is (experimentally) shown to equally proceed mediated by purified enzymes *in vitro* and in cellular extracts.

To shed more light on these intriguing questions, we applied the “calibrated” QM and QM/MM methodology used previously in computational mechanistic studies of zinc(II) hydrolytic enzymes,^21^ notably dizinc glutamate peptidase II.^22^ In this work and previous computational/experimental studies of the enzyme glutamate carboxypeptidase II (GCPII),^23^ we have shown that an accuracy of 2–3 kcal·mol^–1^ in activation energies can be achieved by the combination of the final QM/PCM single-point energies (or QM/COSMO-RS energies, see the Methods section for more technical details) on top of the optimized QM/MM geometries. This is considered a sufficient accuracy to distinguish between several plausible mechanistic scenarios. Capitalizing on this expertise and method calibration, we herein aim to provide a comprehensive mechanistic view of the (rather surprising) double hydrolysis of the oxadiazole HDACis.

Overall, our data reveal that given their high potency and selectivity, the oxadiazole-based HDAC6is are worthy candidates for further investigations; however, additional studies will be required to properly elucidate their ADME/PK properties and physiological degradation routes in living organisms.

## Results

### Fluorinated Oxadiazoles as HDAC6-Specific Inhibitors

**Figure 1:**
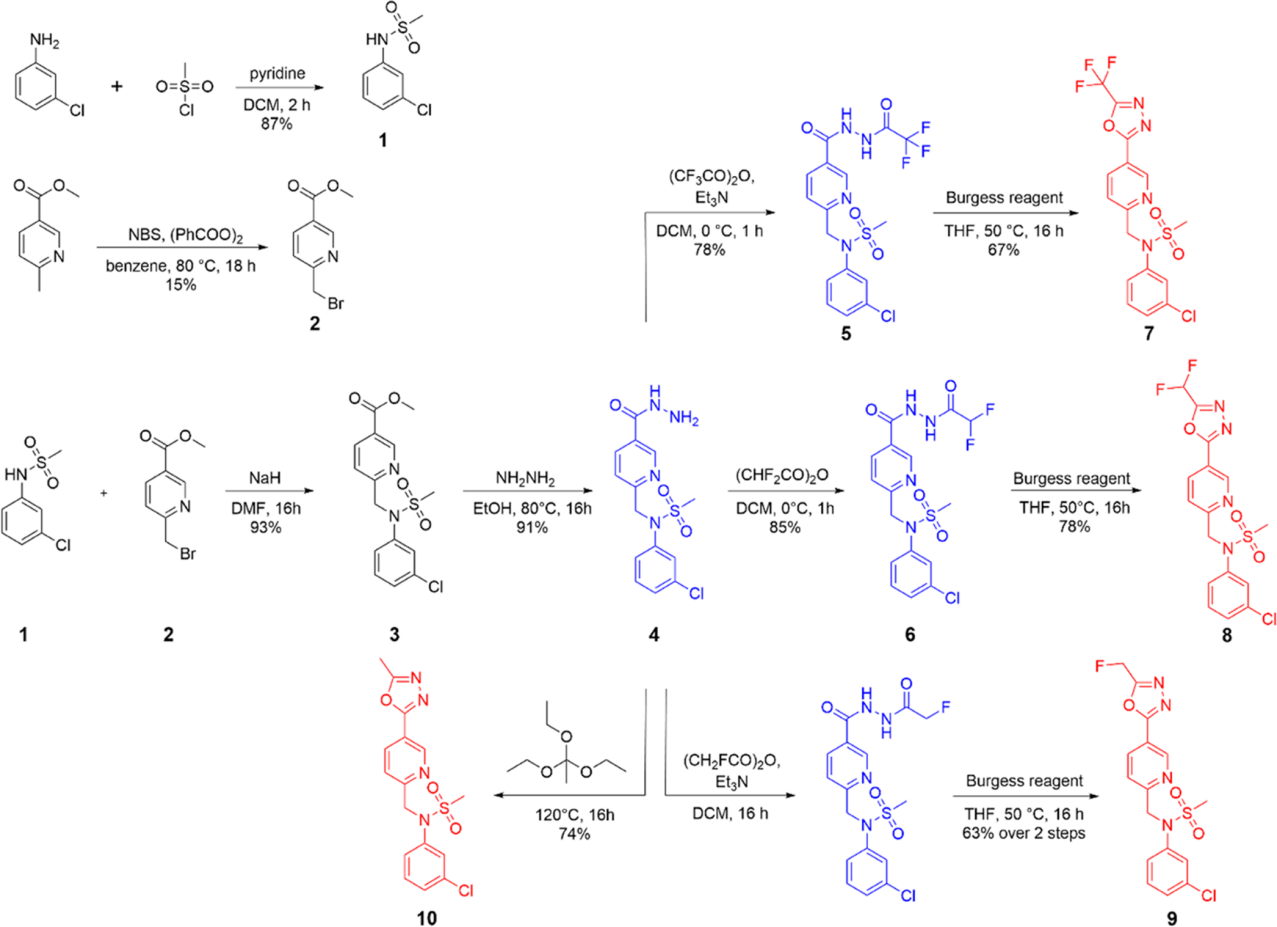
Synthesis of compounds used in the study. Parent inhibitors with an intact substituted 1,3,4-oxadiazole ring are shown in red, while (putative) products resulting from HDAC6 hydrolysis are highlighted in blue.

Functionalized difluoro- and trifluoromethyl-substituted oxadiazoles as HDAC6 inhibitors have been described in the patent literature,^3^ but a more detailed characterization of such compounds, including their HDAC-isoform selectivity and structural characterization, is largely missing, and only recently have some details in this regard been reported.^14,24^ To better assess the importance of the oxadiazole ring substitution by electron-withdrawing fluorinated methyl groups, we first synthesized a series of non-, mono-, di-, and trifluorinated functionalized 1,3,4-oxadiazoles (Figure 1) and evaluated their inhibitory potency against a panel of eleven zinc-dependent human HDACs (Table 1). From this work, we found that the di- and trifluorinated compounds were the most potent inhibitors with IC_50_ values against HDAC6 of 2.1 and 14.6 nM, respectively. Furthermore, both compounds exhibited an exquisite HDAC6-selectivity of over 1000-fold compared to all other HDAC isoforms. Decreased potency was observed for the monofluorinated compound, which exhibited an IC_50_ of 85 nM, while the nonfluorinated methyl analogue failed to show any inhibition even when tested at a concentration of 100 μM.

**Table 1:** *In Vitro* Potency of Compounds Used in This Study

	IC_50_ (nM)						
HDAC	**7**	**8**	**9**	**10**	**4**	**5**	**6**
HDAC1	>20,000^a^	>20,000	NT	NT	>20,000	9166	>20,000
HDAC2	17,410 ± 2814	>20,000	NT	NT	>20,000	>20,000	NT
HDAC3	>20,000	>20,000	NT	NT	>20,000	NT	NT
HDAC4	>20,000	>20,000	NT	NT	>20,000	>20,000	>20,000
HDAC5	>20,000	>20,000	NT	NT	>20,000	NT	NT
HDAC6	14.6 ± 8.0	2.06 ± 0.39	85 ± 2.8	>100,000	2160 ± 392	10,800 ± 3259	14,450 ± 1131
HDAC7	>20,000	>20,000	NT	NT	>20,000	NT	NT
HDAC8	>20,000	>20,000	NT	NT	>20,000	>20,000	>20,000
HDAC9	>20,000	>20,000	NT	NT	>20,000	NT	NT
HDAC10	>20,000	>20,000	NT	NT	NT	NT	NT
HDAC11	>20,000	>20,000	NT	NT	>20,000	NT	NT
zHDAC6 (WT)	NT	NT	NT	NT	3866 ± 1644	NT	41,736 ± 13,638
zHDAC6 (Y745F)	311 ± 150	75 ± 26	>20,000	NT	>50,000	>20,000	>50,000

a IC_50_ values were determined using purified human proteins as described in the Materials and Methods section. NT, not tested; zHDAC6, zebrafish HDAC6, catalytic DD2 domain.

### X-ray Structure of the HDAC6/**4** Complex

**Figure 2:**
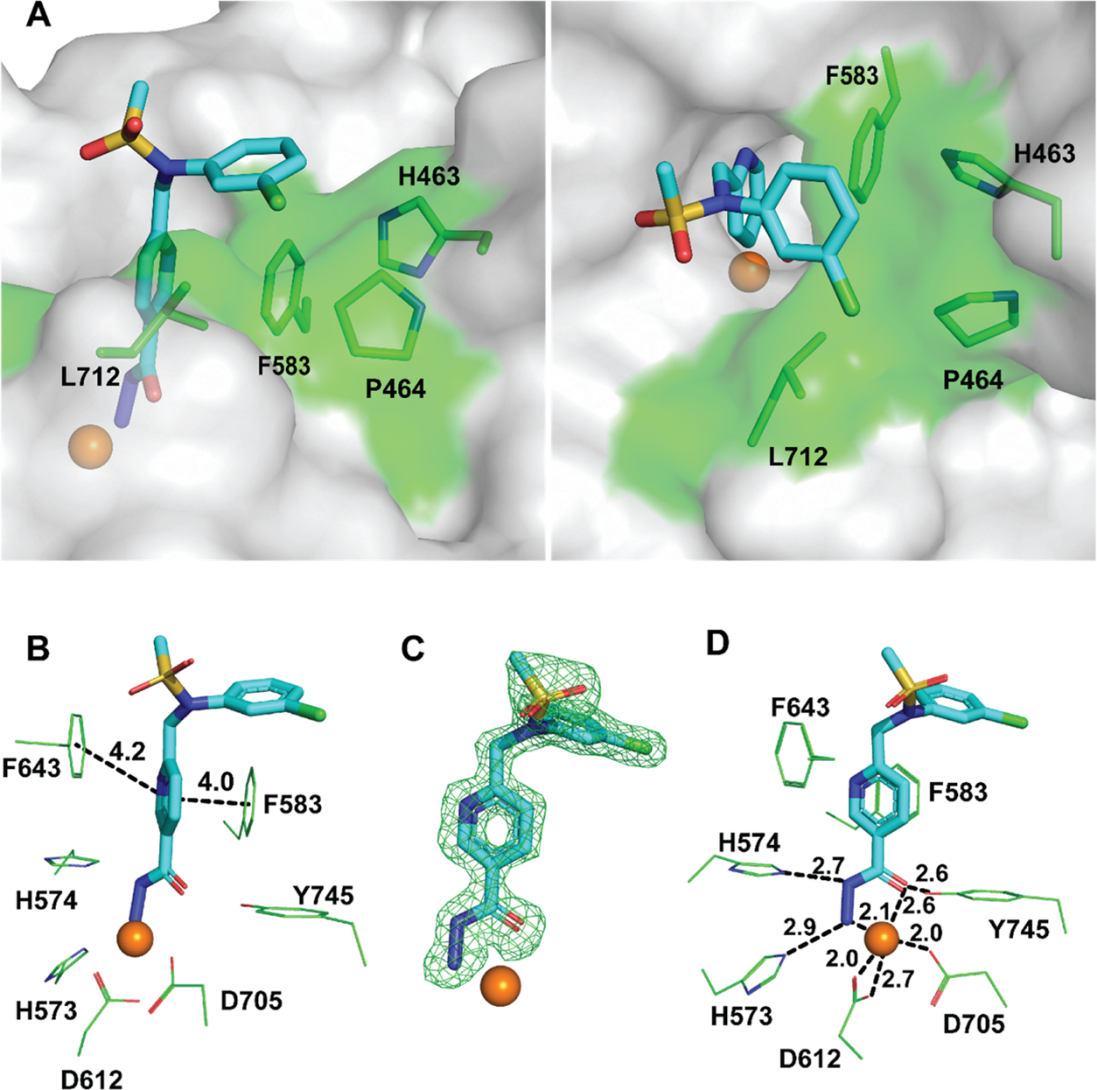
Crystal structure of the zHDAC6/**4** complex (PDB entry 8BJK). (A) Interactions between the chlorophenyl cap and the L1-loop pocket formed by residues H463, P464, F583, and L712 (semitransparent cyan surface representation). (B) π–π stacking interactions between **4** and HDAC6 residues are shown as dashed lines. (C) The *F*_o_–*F*_c_ omit map (green) is contoured at 3.0 σ, and the fitted inhibitor is shown in stick representation. (D) Details of bidentate active-site zinc ion coordination by the hydrazide function. HDAC6 residues are shown as lines with atoms colored green (carbon), red (oxygen), and blue (nitrogen), and the inhibitor is shown in stick representation with atoms colored cyan (carbon), yellow (sulfur), and green (chlorine). The Zn^2+^ ion is shown as an orange sphere. Distances (in Å) are shown as dashed lines.

To elucidate the binding mode of inhibitor **8** in the HDAC6 active site, we co-crystallized **8** with the catalytic DD2 domain of the wild-type zebrafish orthologue (zHDAC6; amino acids 440–798). The crystal structure of the zHDAC6/inhibitor complex was solved by molecular replacement to the resolution limit of 1.35 Å. The chlorophenyl moiety of the cap group of the inhibitor is positioned within van der Waals distances from the side chains of H463, F583, P464, and L712 (modeled in two conformations) of the L1-loop pocket (Figure 2A). One of the more notable features here is the T-shaped π–π stacking interaction between the chlorophenyl group and the side chain of F583 at a distance of 5.1 Å between the aromatic ring centers. At the same time, the methylsulfonyl group is solvent exposed and does not contribute to interactions with the enzyme. The pyridine ring present in the linker engages in parallel π–π stacking with side chains of F583 and F643 lining the entrance tunnel, with distances of 4.0 and 4.2 Å between the respective aromatic residues (Figure 2B). Surprisingly, the *F*_o_–*F*_c_ electron density peaks in the vicinity of the active-site zinc ion revealed that the oxadiazole ring of the parent compound **8** had been hydrolyzed into a hydrazide reaction product (**4**, identified later). Once we had confirmed the chemical nature of the product by liquid chromatography–mass spectrometry (LC–MS)/MS, it was modeled into well-resolved *F*_o_–*F*_c_ positive electron density peaks in the later stages of the refinement (Figure 2C). The hydrazide group of **4** coordinates the active-site Zn^2+^ ion in a bidentate fashion, with an interatomic distance of 2.6 and 2.1 Å between the Zn^2+^ ion and the C=O and the terminal NH_2_ group of the hydrazide, respectively. Furthermore, the C=O group accepts a hydrogen bond from the hydroxyl group of Y745 (2.6 Å), and the N–N group forms hydrogen bonds with the imidazole ring present in the side chains of H573 (2.9 Å) and H574 (2.7 Å; Figure 2D).

### Putative Reaction Scheme and Kinetics of Inhibitor Hydrolysis *In Vitro*

**Figure 3:**
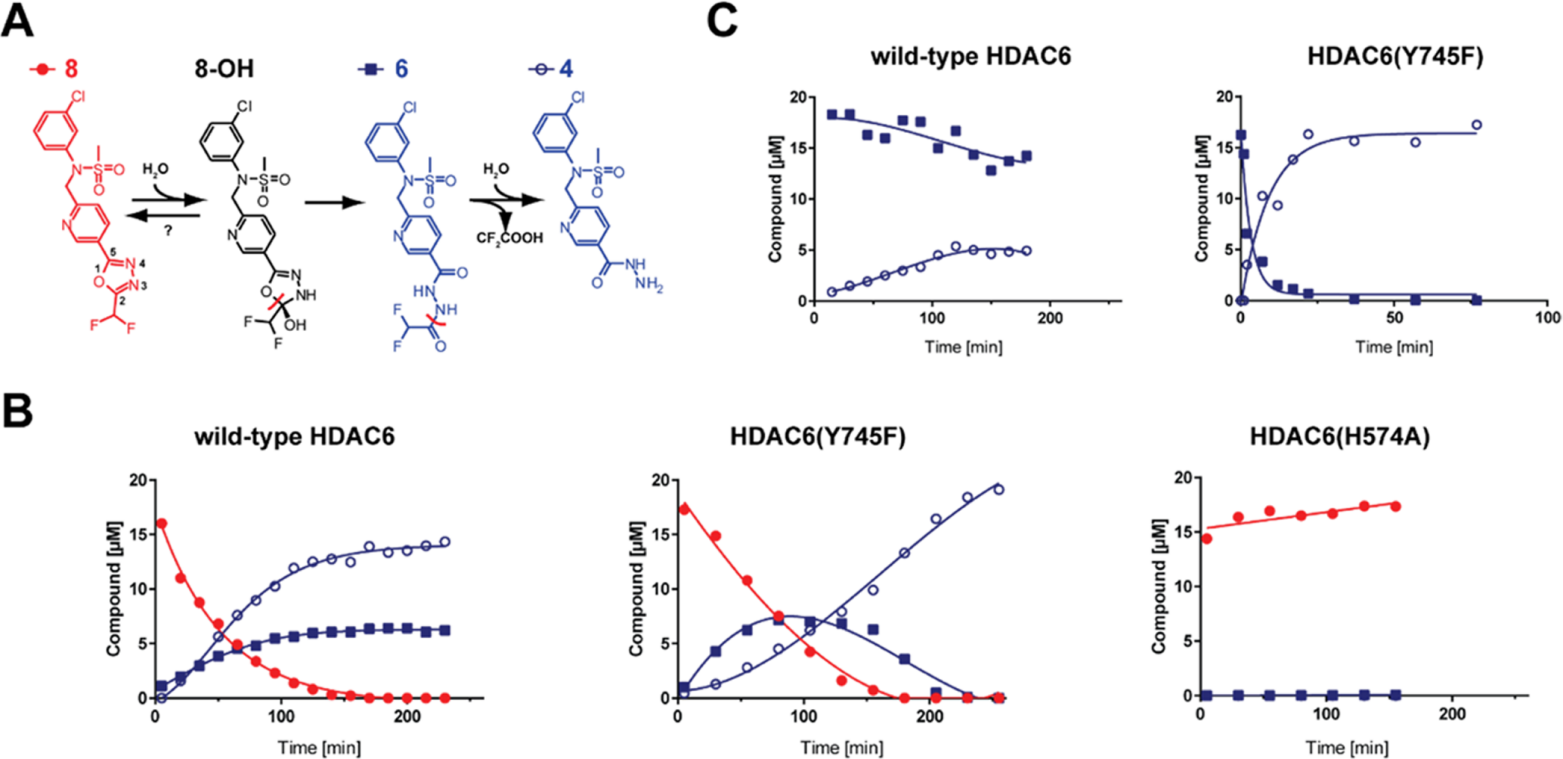
Inhibitor hydrolysis by purified HDAC6 variants *in vitro*. Panel (A): Hydrolysis scheme of **8** by HDAC6. Cleaved bonds are marked by red lines. Panel (B): Kinetics of compound **8** hydrolysis by wild-type HDAC6 and HDAC6(Y745F) and HDAC6(H574A) mutants. Compound **8** (20 μM final concentration) was mixed with 2 μM wild-type HDAC6 in the assay buffer, and reaction mixtures were incubated at 30 °C. Aliquots of reaction mixtures were analyzed by LC–MS/MS at given time points by quantifying concentrations of compounds **4**, **6**, and **8**. Analysis of a putative intermediate **8-OH** was not technically possible as it is unstable in aqueous solutions and thus cannot be synthesized to generate a corresponding analytical method and calibration curve. No hydrolysis of compound **8** was observed for the HDAC6(H574A) mutant. Panel (C): Kinetics of compound **6** hydrolysis by wild-type HDAC6 and the HDAC6(Y745F) mutant. Reaction conditions and quantifications were identical as described for panel (B).

Based on our crystallographic findings, we propose a putative reaction pathway for the conversion of the starting 1,3,4-oxadiazole **8** to the final hydrazide **4**, as shown in Figure 3A. In the first step, the chelation of the oxadiazole ring with the zinc atom present in the active site allows for the facile addition of a water molecule to the C=N group to form the unstable tetrahedral intermediate **8-OH**. This intermediate then undergoes ring opening with cleavage of the C–O bond of the hydroxylated oxadiazole ring to produce the corresponding difluoroacetylated hydrazide. In the next step, the reactive difluoroacetyl group undergoes hydrolytic cleavage to release difluoroacetate and the final monoacylated hydrazide. To confirm our predictions experimentally, the putative reaction products of **8** were synthesized (Figure 1) and used as standards to monitor the hydrolytic reactions *in vitro*. Furthermore, their inhibitory potency against HDAC6 was assayed, and the results are shown in Table 1.

To provide biochemical evidence for the hydrolysis of **8** in solution, we first incubated **8** with the wild-type enzyme and determined time-dependent changes in concentrations of compounds **8**, **6**, and **4** in the reaction mixture using LC–MS/MS. Under assay conditions (20 μM **8**, 2 μM zHDAC6, 30 °C), the inhibitor was fully hydrolyzed in <180 min, with the concomitant increase of the first (**6**) and the second (**4**) products (Figure 3B), while no hydrolysis was observed in the control reaction without the enzyme (not shown) or with the H574A mutant (Figure 3B, third graph)(not shown). Interestingly, upon the near-complete depletion of **8** (at approximately 120 min), the rate of the second transformation (**6**–**4**) is stalled. The decrease in the reaction rate of the second step is even more surprising, given the absence of the original inhibitor along with the increase in the concentration of the intermediate, as both of these shall accelerate the final reaction step (Figure 3B, the first graph).

To assess the importance of HDAC6 residues, known to be involved in the deacetylation of peptide substrates, we quantified the hydrolysis of **8** by employing the Y745F and H574A mutants that were shown to be catalytically inactive on acylated peptides (Figure 3B).^16,17^ Interestingly, **8**/**6** were also hydrolyzed by the HDAC6(Y745F) mutant, which is “catalytically-dead” on peptide substrates, yet with markedly different kinetics compared to the wild-type enzyme. While the rate of hydrolysis of **8** by the HDAC6(Y745F) mutant was comparable to the wild-type HDAC6, the second hydrolytic reaction from **6** to **4** proceeded with different kinetics. For the wild-type enzyme, the initial fast reaction rate was followed by the stalled “second” phase. In contrast, for the Y745F mutant, the initial slow phase was followed by a second “burst” phase upon depletion of **8**. These findings were corroborated by the simplified reaction system, where the kinetics of the “isolated” second reaction was evaluated by following the hydrolysis of **6** as the “substrate” by wild-type HDAC6 and its HDAC6(Y745F) mutant (Figure 3C). Finally, the H574A mutant, where the general-base histidine is mutated, revealed no catalytic activity against any tested compounds (Figure 3B).

### Hydrolysis of **8** in Cell Lysates

**Figure 4:**
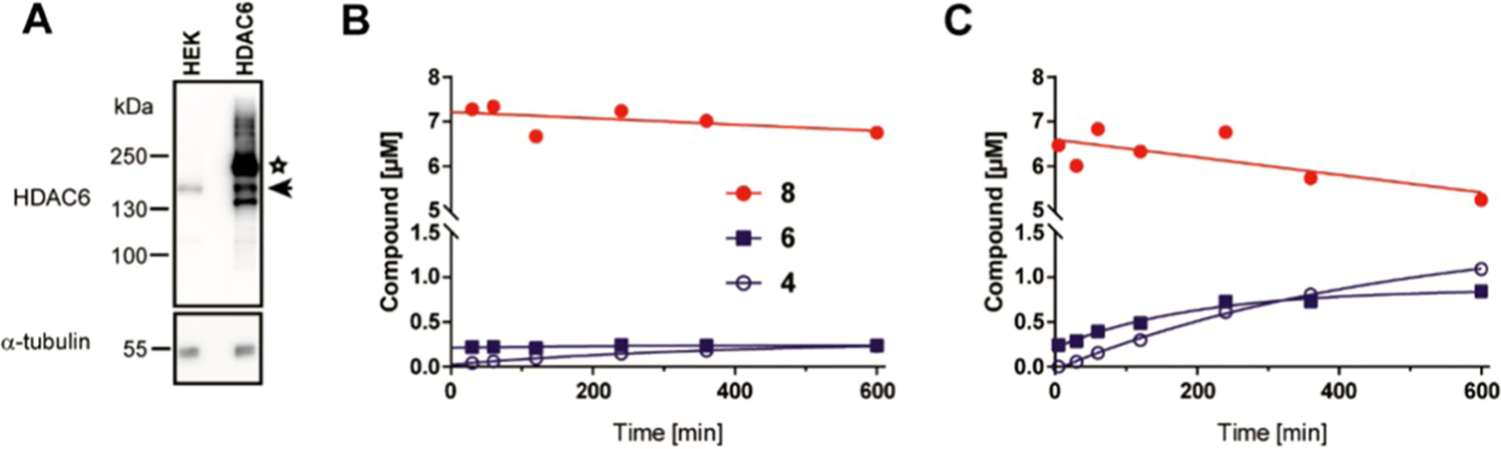
Inhibitor transformation in cell lysates. Panel (A): Western blot analysis of HDAC6 expression in parent HEK293T cells (HEK) and HEK293/HDAC6 (HDAC6) stable transfectants. A-tubulin was used as a loading control. The asterisk and arrow mark the HALO-HDAC6 fusion and endogenous HDAC6, respectively. Panels (B, C): Kinetics of the hydrolysis of **8** in lysates of HEK293T (B) and HEK293/HDAC6 (C) cells. Compound **8** (20 μM final concentration) was mixed with cell lysates (final protein concentration of 2.5 mg mL^–1^), and compound hydrolysis at 37 °C was monitored by LC–MS/MS upon inhibitor extraction by acetonitrile precipitation.

Additionally, to assess whether **8** is modified in more relevant physiological settings, we further evaluated the hydrolysis of **8** in HE293 cell lysates as monitored by LC–MS/MS. HEK293 and HEK293 cells stably transfected with human HDAC6 (HEK293/HDAC6) were used to mimic cell lines and tissues expressing low and high levels of HDAC6, respectively. Both cell lines hydrolyzed **8** to **6** and **4** as observed *in vitro.* The hydrolytic reaction was more efficient using the HEK293/HDAC6 lysate due to higher expression levels of HDAC6 (Figure 4). Our cellular data thus clearly confirm that the observed hydrolysis is not an artifact of our *in vitro* assay and is likely to occur in animal/human tissues upon inhibitor treatment.

### QM/MM Characterization of 1,3,4-Oxadiazole Hydrolysis by HDAC6

We employed QM/MM and QM calculations to provide detailed mechanistic insights into the hydrolysis of **8** by HDAC6, including the structural characterization of the most likely reaction coordinates and corresponding energy barriers of the individual elementary steps. For the sake of clarity, we first present the most likely reaction coordinate as predicted computationally, whereas alternative pathways and structural alternatives will be briefly mentioned in the next section and reported in more detail in the Supporting Information (SI), including three-dimensional (3D) coordinates of all structures studied. For the same reason (clarity), we will present only one set of computed energies, namely, the *G*′_comp_ = *E*(QM(TPSS-D3/def2-TZVP)/COSMO(ε_r_ = 8))//QM/MM + *E*_ZPVE_ + *RT* ln *Q* + *RT*. These represent the single-point energy computed at the DFT(TPSS-D3) level in a polarized continuum (ε_r_ = 8) at the optimized QM/MM geometries, corrected for the entropic effects obtained via normal-mode analysis (see Computational Details). This is essentially identical to the previously experimentally calibrated protocol for the GCPII hydrolysis.^22^

#### Michaelis Complex (**MC**_**S1**_)

**Figure 5:**
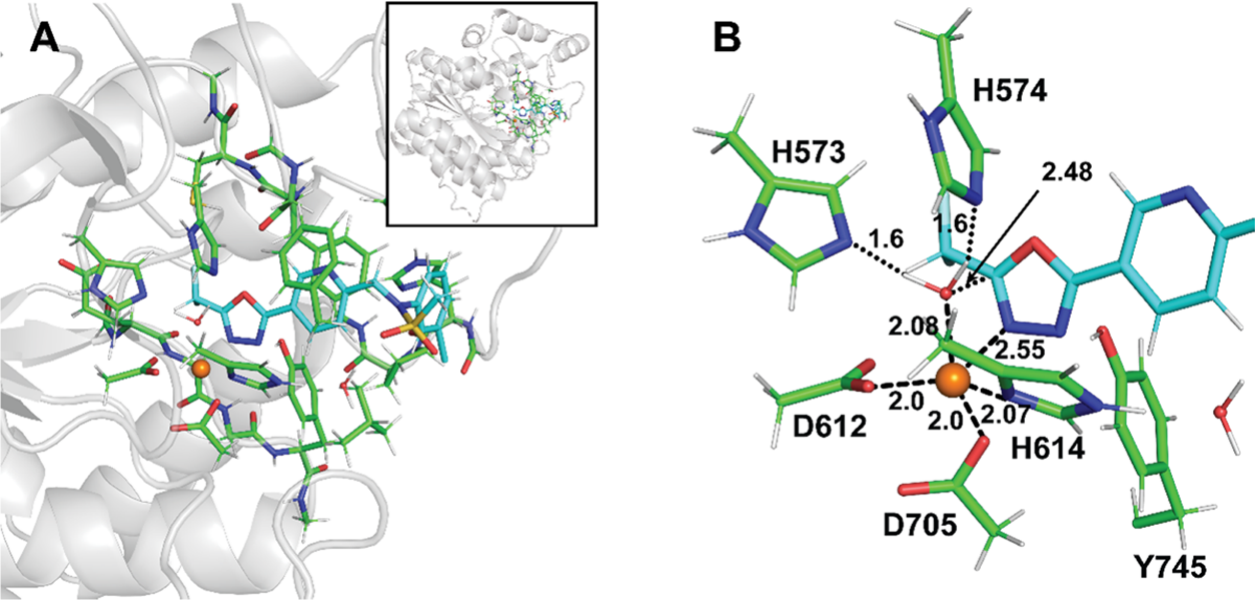
QM/MM model. Panel (A): The full-size QM system (∼320 atoms) used in the QM/MM and subsequent QM single-point energy calculations is shown in stick representation with carbon atoms of HDAC6 and compound **8** colored green and cyan, respectively. Hydrogens were omitted for clarity in this panel. Panel (B): Details of the HDAC6/**8** complex (Michaelis complex, denoted as **MC**_**S1**_) with hydrogen atoms displayed.

The lowest energy structure of the HDAC6/**8** complex is depicted in Figure 5, and it is, in essential features, similar to the model of the Michaelis complex presented by Cellupica and colleagues.^24^ Here, the active-site zinc ion is tightly coordinated by the side chains of D612, D705, and H614 featuring standard M-X distances: *R*(Zn-O_D612_) = *R*(Zn-O_D705_) = 2.00 Å, and *R*(Zn-N_H614_) = 2.07 Å, respectively. Furthermore, the oxadiazole ring of the inhibitor is loosely coordinated to the zinc ion via its N_3_ nitrogen at *R*(Zn···N_3_) = 2.55 Å. The Zn(II) coordination sphere is then completed by the catalytic water placed at *R*(Zn-O_w_) = 2.08 Å. The five ligands adopt distorted trigonal bipyramidal geometry (with H_2_O and D705 as axial ligands). In this configuration, the water molecule is well positioned for the nucleophilic attack on the C_2_ carbon atom of the oxadiazole ring (*R*(C_2_···O_W_) = 2.48 Å), and its two hydrogens are H-bonded to N_ε_ nitrogens of the {His}_2_ dyad comprising H573 and H574. We note in passing that the His dyad is a structural hallmark of the active site of all human HDACs.^25,26^ In our calculations, we postulate that both histidines are neutral in the Michaelis complex (**MC**_**S1**_) and thus perfectly suited to act as hydrogen-bond acceptors. This is in line with a previous suggestion^27^ and in minor contrast to the work of Prejano et al.,^20^ where authors considered one of the His to be protonated in their **MC** structure. Moreover, our assignment of initial protonation states is also compatible with the earlier work of Christianson and co-workers,^16^ and in our opinion, it better corresponds to experimental kinetic measurements carried out in this work at pH = 7.4. At the same time, we acknowledge that both general acid and general-base mechanisms (referring to the initial catalytic step) might be operational at varying pH ranges.

#### First Hydrolytic Step: from the **MC**_**S1**_ to **P**_**S1**_ (**6**)

**Figure 6:**
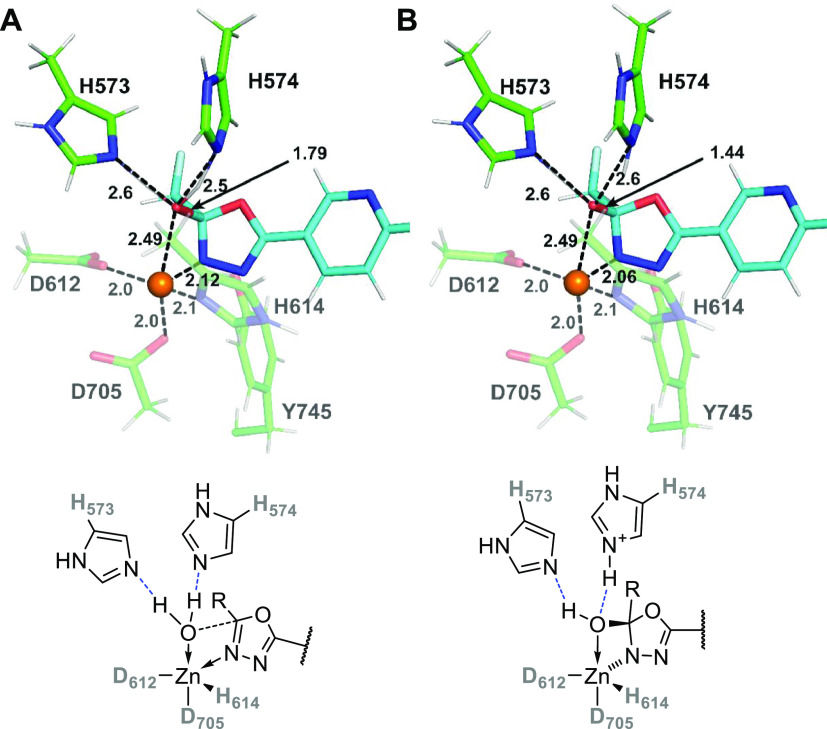
QM/MM equilibrium structures corresponding to A/**TS1**_**S1**_ and B/**TI**_**S1**_.

In the first hydrolytic step (step 1, S1), which results in the opening of the inhibitor oxadiazole ring, there is a straightforward path from **MC**_**S1**_ via the transition state **TS1**_**S1**_ to the tetrahedral intermediate **TI**_**S1**_ (Figure 6). The Δ*G*′_comp_ values (cf. Figure 10) along the reaction coordinate (set at 0.0 kcal·mol^–1^ for **MC**_**S1**_) are 4.4 and −2.7 kcal·mol^–1^ for **TS1**_**S1**_ and **TI**_**S1**_, respectively, pointing toward a very facile reaction step with overall energetics only slightly exergonic. At the **TS1**_**S1**_, the key C–O distance/bond is *R*(C_2_-O_W_) = 1.79 Å (cf. Figure 6). The Zn-O_W_ coordination bond is elongated to *R*(Zn-O_W_) = 2.49 Å, and the water molecule preserves its two strong hydrogen bonds with the H_573/574_ dyad. The oxadiazole′s N_3_ nitrogen moves closer to the Zn(II) ion (*R*(Zn-N_3_) = 2.12 Å), which suggests tighter coordination. In the **TI**_**S1**_ intermediate (Figure 6B), the *R*(Zn-N_3_) distance is further shortened to 2.06 Å; the C_2_ carbon of the oxadiazole ring assumes sp^3^-hybridization as a result of the C_2_-O_W_ bond formation.

Proceeding along the reaction coordinate, our QM/MM simulations suggest that the pathway from **TI**_**S1**_ through **TS2**_**S1**_ to the first hydrolytic product **P**_**S1**_ seems to be the most intriguing and potentially rate-determining elementary reaction step.

**Figure 7:**
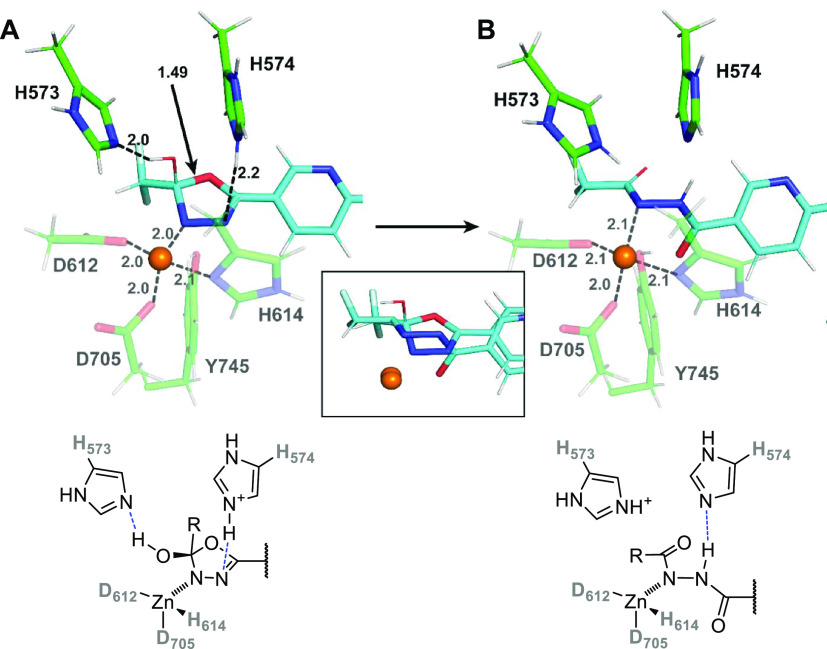
QM/MM equilibrium structures corresponding to (A) **TS2**_**S1**_ and (B) **P**_**S1**_. The rotation of the cleaved oxadiazole ring along the C–C bond connecting the oxadiazole moiety with the rest of the inhibitor can be clearly seen; it occurs shortly after **TS2**_**S1**_.

The QM/MM transition state (**TS2**_**S1**_) is depicted in Figure 7, together with the energetically most stable product of the first hydrolysis, **P**_**S1**_. The **TS2**_**S1**_ energy is computed to be Δ*G*′_comp_^‡^ = 18.3 kcal·mol^–1^ (i.e., an activation energy of 18.3 + 2.7 = 21 kcal·mol^–1^ is needed to proceed from the **TI**_**S1**_ to the **P**_**S1**_ through **TS2**_**S1**_). We also postulate that the **TI**_**S1**_ → **TS2**_**S1**_ → **P**_**S1**_ transformation is associated with a substantial conformational change that involves rotation of the (cleaved) oxadiazole ring by approximately 180° (cf. Figure 7A,B and the inset figure). According to our QM/MM calculations, this happens shortly after **TS2**_**S1**_ at the *R*(H_W/His+_···N_4_) < 2.0 Å (see below for details), where H_W/His+_ denotes the proton that was originally on the catalytic water and ended up on H574 after **TS1**_**S1**_. It shall be noted that such rotation must occur at some point along the reaction pathway in order to converge into the experimentally determined structure of the final product discussed below (where the two nitrogens are on the opposite site compared to the putative reactant structure).

As also shown in Figure 7, at *R*(H_W/His+_···N_4_) = 2.2 Å, corresponding to the highest point on the one-dimensional (1D) scan of the proton transfer from H574 to the N_4_ nitrogen of the oxadiazole, the breaking C_2_–O_1_ bond elongates from 1.44 to 1.49 Å. Further shortening of the H_W/His+_···N_4_ distance is already energetically downhill (e.g., 4–5 kcal·mol^–1^ for *R*(H_W/His+_···N_4_) = 2.1 Å), and a major conformational change, spontaneously leading to **P**_**S1**_, occurs at *R*(H···N_oxadiazole_) = 1.9 Å. The Δ*G*′_comp_ of **P**_**S1**_ is −15.0 kcal·mol^–1^ (with respect to the initial **MC**_**S1**_), which represents a huge thermodynamic driving force for the first hydrolytic step. As can be seen in Figure 7, the **P**_**S1**_ (HDAC6/**6**) complex is characterized by close-to-tetrahedral coordination of the active-site Zn^2+^ ion by side chains of D612, D705, and H614, and the N_3_ nitrogen atom of the “original” oxadiazole ring of the inhibitor. The H_573/574_ pair, unlike in the **MC**_**S1**_ structure, acts as both a hydrogen-bond donor (H_573_) and an acceptor (H_574_). At this point, the role of Y745 can also be highlighted, at least in qualitative terms. It acts as the hydrogen-bond donor in all steps of the first hydrolysis, first to donate the hydrogen bond to the N_4_ atom of the oxadiazole ring and later to the O_1_ atom of the cleaved ring.

#### Second Hydrolytic Step: from **P**_**S1**_ to **P**_**FIN**_ (**4**)

The second hydrolytic step requires one more water molecule in the active site. As for the “catalytic water” for this second step, we may speculate that a second-sphere water molecule present in the original **MC**_**S1**_ complex (cf. Figure 5) may easily replace the water consumed in the first catalytic step and act as the catalytic water in the second step. Alternatively, a water molecule added from the solution can also migrate to the active site and become the catalytic water. To estimate the energy balance accompanying its coordination/addition and to connect reaction coordinates for the first and second hydrolytic steps (which differ by one water molecule), we attempted to calculate the binding free energy of the addition of a water molecule to **P**_**S1**_, resulting in one of the **MC**_**S2**_ alternatives considered below. We employed the experimentally (spectroscopically) calibrated protocol recently applied in studying the hydration of the active site in the binuclear copper tyrosinase *oxy-*form (*oxy*-Ty).^28^ The protocol is based on the COSMO-RS solvation model and includes the *E*_ZPVE_*+ RT* ln* Q* + *RT* entropic and thermal energy corrections, which are of paramount importance for processes studied here: the addition of water molecule from the solvent to a confined enzyme active site (see Methods for details of the calculation). The computed free energy change for the reaction **P**_**S1**_ + H_2_O → **MC**_**S2**_^**gm**^ (global minimum found for the Michaelis complex for the second hydrolytic step discussed) is Δ*G*_CRS_ = −1 kcal·mol^–1^; admitting that the error in this free energy value can be somewhat larger (presumably 2–3 kcal·mol^–1^) than in relative Δ*G*′ values along the individual reaction coordinates. Still, it can be, in our opinion, safely concluded that the binding of the additional water needed for the hydrolytic step 2 (S2) is quite close to ergoneutral. Computing the Δ*G*_CRS_ (**P**_**S1**_ + H_2_O → **MC**_**S2**_^**gm**^) energy affords a connection between the two potential energy surfaces for steps S1 and S2 (cf. Figure 10). Both steps can be then related to the “0.0” energy of **MC**_**S1**_ (i.e., Δ*G*′_comp_ of **MC**_**S2**_^**gm**^ is therefore −15.0 kcal·mol^–1^ of **P**_**S1**_ minus −1 kcal·mol^–1^ for the reaction **P**_**S1**_ → **MC**_**S2**_^**gm**^, resulting in a value of −16.0 kcal·mol^–1^ with respect to **MC**_**S1**_, which thus defines an “absolute” scale).

**Figure 8:**
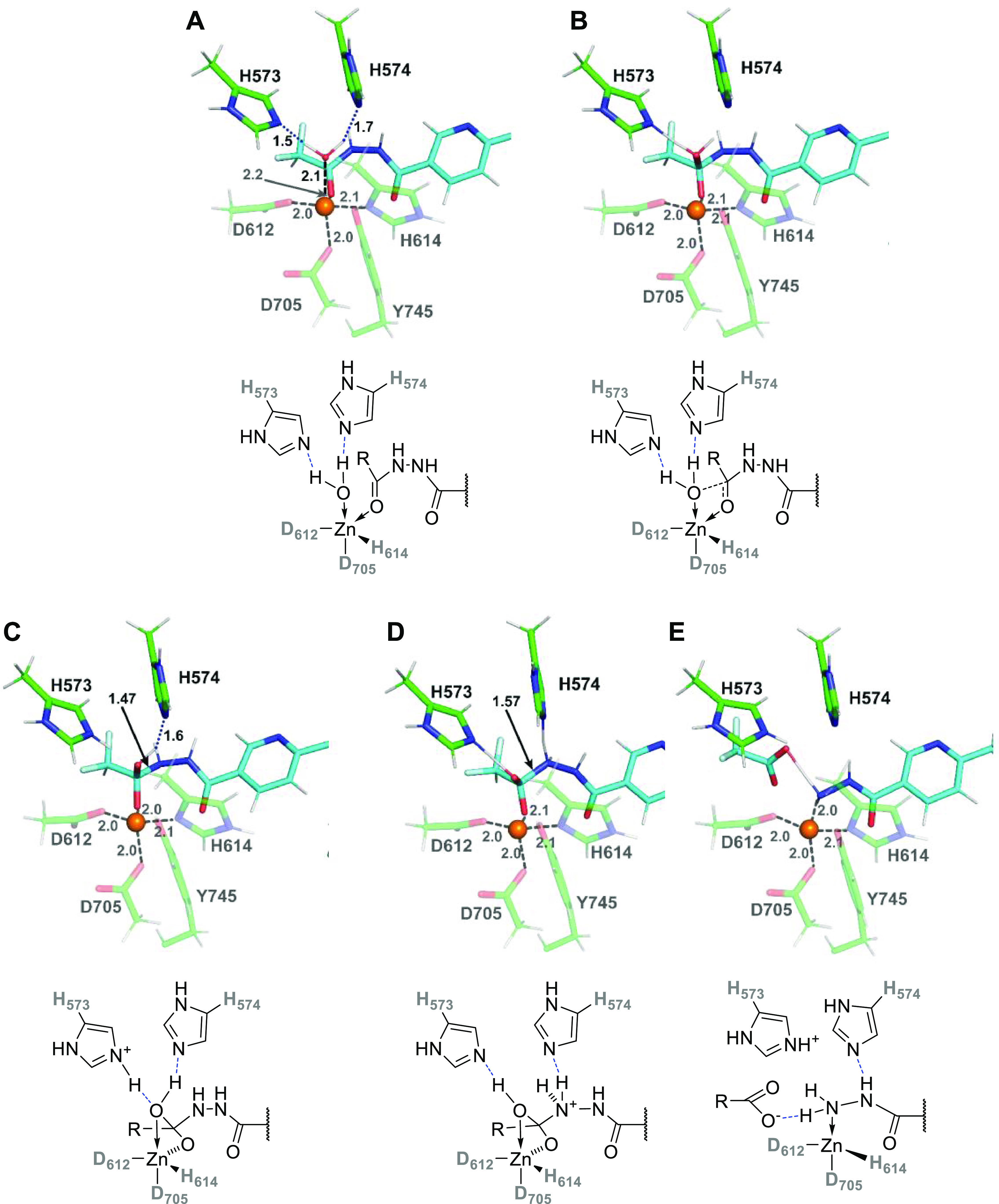
QM/MM equilibrium structures corresponding to (A) **MC**_**S2**_′, (B) **TS1**_**S2**_, (C) **TI**_**S2**_, (D) **TS2**_**S2**_, and (E) **P**_**S2**_.

As stated above, we tested various structural alternatives of **MC**_**S2**_ (all structures are deposited in the Supporting Information). The two energetically lowest structural alternatives feature tetrahedral coordination of the active-site Zn^2+^ ion, essentially replicating the **P**_**S1**_ complex, with the added water molecule positioned in the second coordination sphere. However, extensive QM/MM and QM calculations did not reveal any energetically plausible pathway from any of these two structures (that do not have the activated, zinc-coordinated water), leading to the **TS1**_**S2**_ and **TI**_**S2**_. The only viable reaction pathway discovered computationally is via the coordination of the water molecule to the zinc ion and coordination of the ligand via the oxygen atom (**MC**_**S2**_′ depicted in Figure 8A). This is associated with a free energy cost of 12.2 kcal·mol^–1^. We assume that most of this energy cost comes from the change of the coordination of the (semi-hydrolyzed) substrate (cpd **6**) from tight coordination by nitrogen to looser coordination by carbonyl oxygen. Interestingly, such a coordination mode (**MC**_**S2**_′) is almost identical to structures of Michaelis complexes of HDAC6, with its natural ligands comprising an *N*-acetylated-lysine moiety, which has been studied by others.^20,27^

A can be seen in Figure 8B,C, there is then an analogous pathway to **TS1**_**S2**_ and **TI**_**S2**_, as described above for the first (S1) hydrolytic step. This includes (i) the His dyad playing the role of hydrogen-bond acceptors (the above-mentioned de-coordination of the N_3_ atom of the cleaved oxadiazole ring upon the **MC**_**S2**_^**gm**^ → **MC**_**S2**_′ transition leads exactly to the “protonation state” of ligand **6**, as depicted in Figure 3A, and thus, both His are in their neutral states prior to the S2 hydrolysis, ready to act as H-bond acceptors); (ii) ideal orientation of the coordinated water molecule for the nucleophilic attack, including the favorable C···O_W_ distance; and (iii) a comparable activation energy for the appearance of the **TI**_**S2**_ intermediate relative to **MC**_**S2**_′, Δ*G*′_comp_^‡^ (**MC**_**S2**_′ → **TS1**_**S2**_) = 5.8 kcal·mol^–1^, which translates to a value of 18.2 kcal·mol^–1^ from **MC**_**S2**_^**gm**^ and to 2.2 kcal·mol^–1^ (on the “absolute scale”). The energy of **TI**_**S2**_ is then −3.4 kcal·mol^–1^ (on the absolute scale). The pathway to the final product **P**_**S2**_ via **TS2**_**S2**_ (Figure 8D,E) is relatively straightforward and involves proton transfer from H574 to the N_3_ nitrogen of the ligand and cleavage of the C_2_–N_3_ bond. At the **TS2**_**S2**_, the proton is almost shared between H574 and the ligand, whereas the C_2_–N_3_ bond is elongated from 1.47 to 1.57 Å. The Δ*G*′_comp_^‡^(**TS2**_**S2**_) is 5.9 kcal·mol^–1^ (absolute scale), which translates to an activation energy of 9.3 kcal·mol^–1^ with respect to **TI**_**S2**_. The Δ*G*′_comp_ of (**P**_**S2**_) is −8.3 kcal·mol^–1^. Again, this represents an ample thermodynamic driving force for the second hydrolytic step S2.

**Figure 9:**
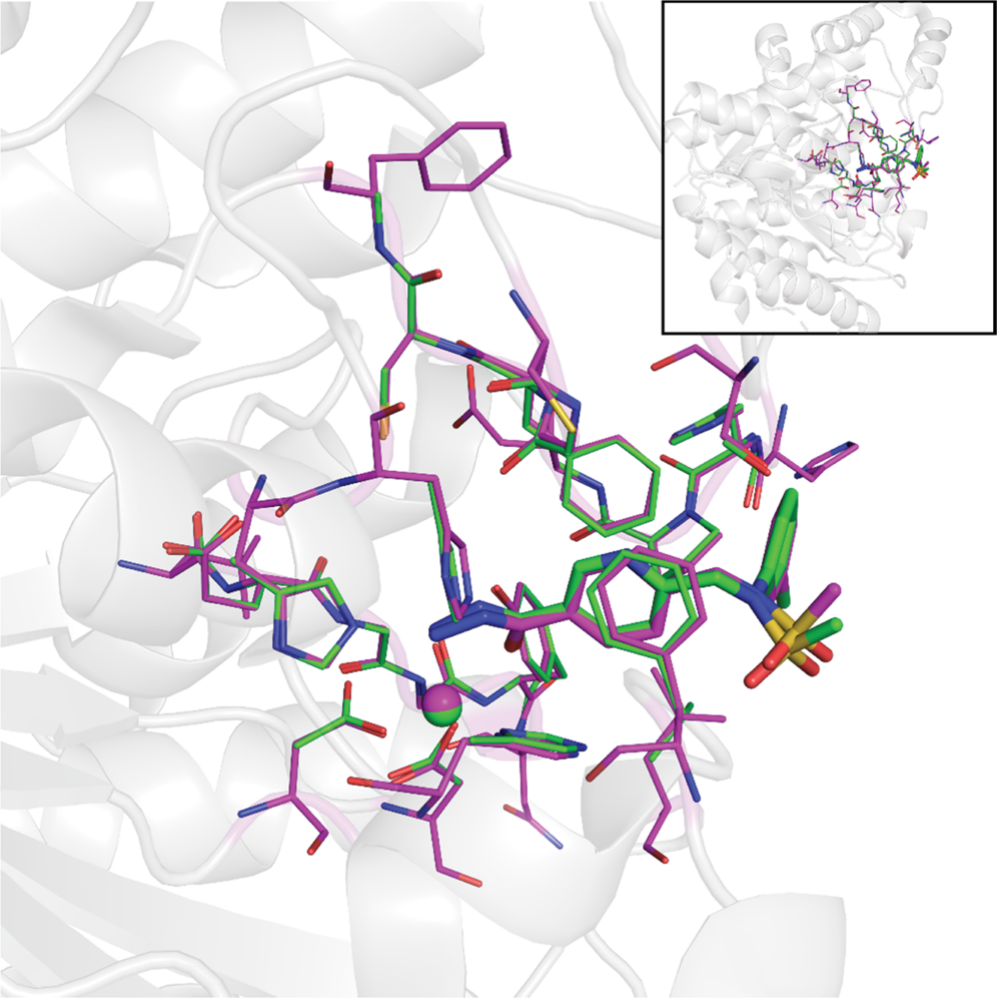
Superposition of the experimental (X-ray; green carbon atoms/zinc ion) and computed (equilibrium QM/MM; cyan carbon atoms/zinc ion) structures of the product of the double hydrolysis of **8** by HDAC6.

The experimental structure of **PS**_**2**_ (HDAC6/**4**) reported in this work suggests that the difluoroacetate moiety leaves the active site prior to the potential release of the remaining part of the structure of **8** (i.e., the moiety **4**). Employing the same protocol as for estimating the free energy of water binding at the beginning of the second hydrolytic step (vide supra), we calculated the free energy of dissociation of difluoroacetate from the active site to be −12.0 kcal·mol^–1^. The final QM/MM structure of the **P**_**S2**_ with difluoroacetate dissociated (denoted **P**_**FIN**_) is an excellent anchor point to correlate the computed and experimental structures. As can be seen in their superposition in Figure 9, the agreement is truly excellent. It shall be re-emphasized that the **P**_**FIN**_ QM/MM structure was obtained through a relatively complicated reaction pathway starting from **MC**_**S1**_ (putative binding mode of **8**) and, therefore, we consider the excellent structural agreement between HDAC6/**4** and the **P**_**FIN**_ an *a posteriori* verification of the proposed reaction mechanism.

#### Alternative Pathways

In Table S1, we summarized the most important alternative pathways and the corresponding equilibrium or transition state structures that were computationally studied. Some of them may represent nonintuitive or unlikely alternatives, but we studied them for the sake of completeness. The corresponding three-dimensional model structures can be found in the Supporting Information as well.

In brief, we tested the following hypotheses: (1) reverse orientation of the oxadiazole ring in the **MC**_**S1**_ complex; (2) various protonation states for **TI**_**S1**_ and **P**_**S1**_ (cf. Table S1 for computed energies); (3) various two-dimensional (2D) scans to assess the validity of fairly complicated **TS2**_**S1**_ at the QM/MM level; (4) involvement of Y745 in proton-transfer steps, notably in **TS2**_**S2**_. (5) various bonding modes of water to obtain **MC**_**S2**_^**gm**^ (global minimum); and (6) various activation pathways for the second hydrolytic steps (competing with the **MC**_**S2**_′ structure). None of these efforts did identify energetically feasible pathways other than the one reported above.

## Discussion

In this report, we show that fluorinated 1,3,4-oxadiazoles are potent and highly selective inhibitors of HDAC6, yet, at the same time, these compounds are degraded by HDAC6 in a substrate-like manner, yielding the corresponding hydrazide products. Interestingly, hydrazide-based compounds were developed as HDAC inhibitors in the past, yet they typically have over 1000-fold lower potency compared to inhibitors with other ZBGs.^9,10,29−31^ Similar potency differences are also observed in the present manuscript. First, di- and trifluorinated oxadiazoles **8** and **7** are approximately 1000- and 150-fold more potent, respectively, compared to the hydrazide product **4**, pointing toward lower effectivity of the hydrazide function in engaging the active-site zinc of HDACs. Similarly, the hydroxamate-based analogue of **8** and **4**, which was reported previously,^32^ is approximately 6- and 6200-fold more potent against HDAC6 when compared to the potency of **8** and **4**, respectively. At the same time, replacement of the oxadiazole function by hydroxamate results in markedly decreased inhibitor selectivity, exemplified by the HDAC6/HDAC1 selectivity index,^32^ confirming the critical importance of the oxadiazole warhead to the inhibitor isoform selectivity.

At first sight, 1,3,4-oxadiazoles do not appear to constitute much of a metabolic liability. Indeed, such 5-membered heterocyclic ring systems are considered to be bioisosteres of ester groups that have been developed to generate analogues that are resistant to hydrolysis by esterases.^33^ Consequently, 1,3,4-oxadiazole-based compounds might be expected to have improved pharmacokinetic parameters (e.g., low clearance and high bioavailability) compared to their hydroxamate-based counterparts. At the same time, oxidative ring opening to form a 1,2-diacylhydrazine has been observed in the case of a 5-lipoxygenase inhibitor, and this transformation is believed to proceed through cytochrome P450-mediated epoxidation of one of the ring′s C=N double bonds.^34^ Furthermore, the opening of the 1,3,4-oxadiazole ring can also result from the action of other enzymes as exemplified by HDACs.^24^ In either case, hydrazides, the oxadiazole degradation products, can be toxic by themselves or can be further metabolized to genotoxic/mutagenic compounds, such as hydrazines.^35^ Taken together, these factors need to be considered when assessing the safety of novel oxadiazole-based HDAC inhibitors.

**Figure 10:**
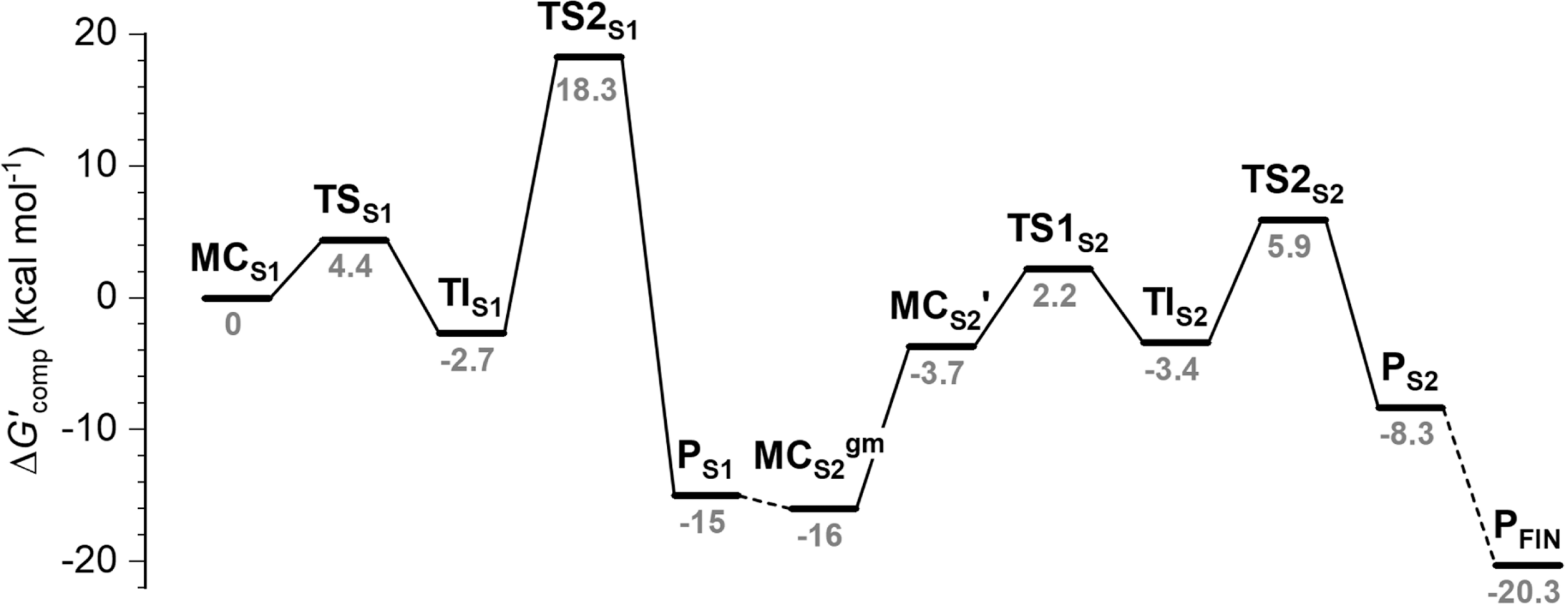
Energetic profile of the two-step hydrolysis of **8** through **6** to **4** catalyzed by HDAC6. Δ*G*′_comp_ is *E*(QM(TPSS-D3/def2-TZVP/COSMO(*ε*_r_ = 8))//QM/MM + *RT*ln*Q* + *RT*). The energetic profile was modeled for three different QM/MM systems: **MC**_**S1**_–**P**_**S1**_, **MC**_**S2**_^**gm**^–**P**_**S2**_, and **P**_**FIN**_ (which are connected by dashed lines via the computed binding free energy of a water molecule on going from **P**_**S1**_ to **MC**_**S2**_^**gm**^ and dissociation of the difluoroacetate anion going from **P**_**S2**_ to **P**_**FIN**_.). All values are in kcal·mol^–1^.

**Scheme 1:**
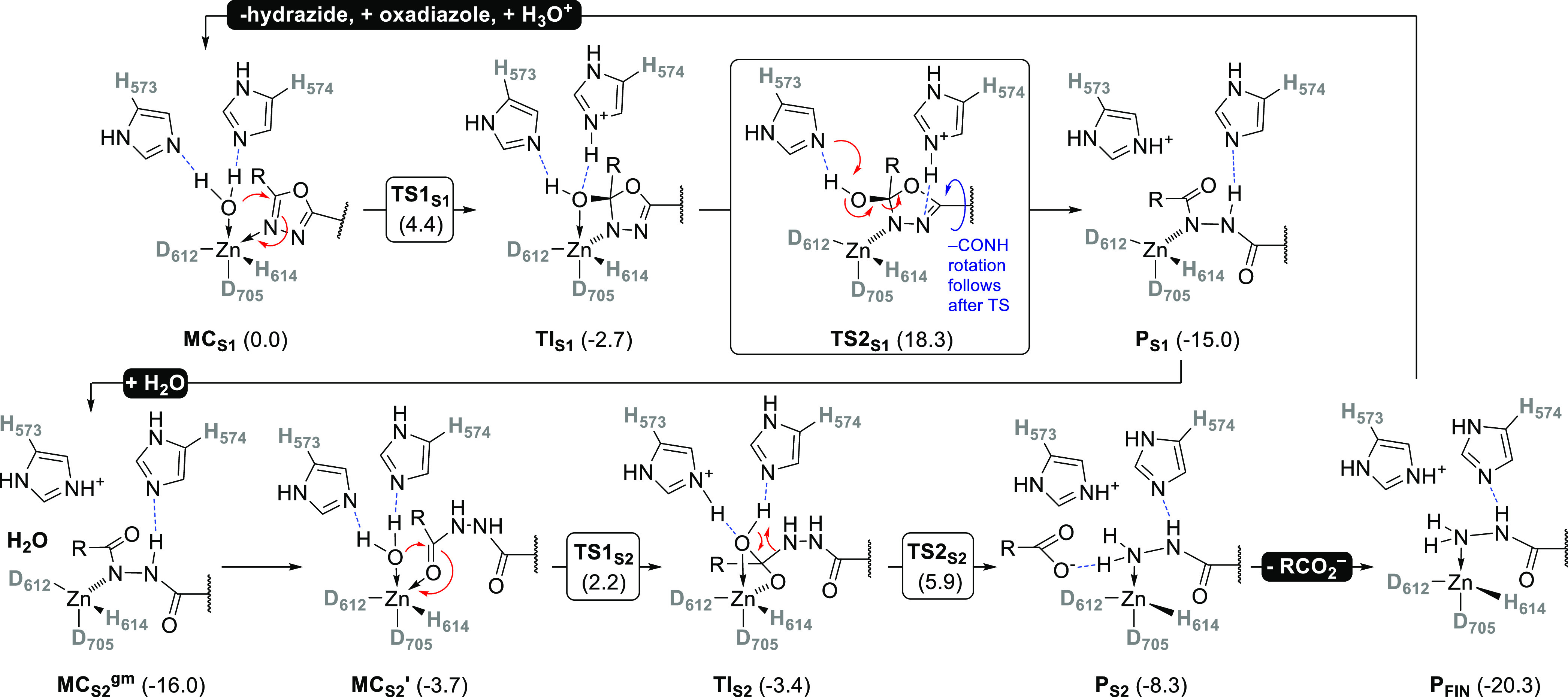
Comprehensive Mechanistic View of the Oxadiazole Hydrolysis by the HDAC6 The computed Δ*G*_comp_^′^ values (in kcal·mol^–1)^ for intermediates and transition states are depicted as well.

To fully understand why HDAC6 is capable to convert oxadiazoles into hydrazides in two hydrolytic steps, we have employed QM/MM and QM calculations. In our recent computational studies of various metalloenzymes (GCPII,^22^ tyrosinase,^28^ Δ^9^-desaturase,^45^ and others), we fine-tuned our computational protocols by directly correlating the calculated data with experimental results. For example, rate constants of various GCPII mutants correlated almost quantitatively with the computed activation energies or the hydration level of the oxy-Ty active site as confirmed by computed free energies of the addition of water molecules correlated with resonance Raman spectroscopic data. This gives us high confidence in the values reported herein. Still, as shortly presented below, we tested the robustness of the Δ*G*′_comp_ values reported above by employing various flavors to the computational protocol (change of the functional, change of the solvation model, size of the active-site model), and it can be concluded that the energy profile depicted in Figure 10 is numerically stable and can be used to draw conclusive computational evidence. The overall reaction mechanism is summarized in Scheme 1.

Both hydrolytic steps are characterized by (what we consider) textbook views of the hydrolysis by zinc(II) enzymes. The presence of a proton shuttle(s), acting as proton acceptors first and donors later, facilitates the binding of a water molecule to the zinc(II) ion, which becomes hydroxide either prior (GCPII) or during (HDAC6) its nucleophilic attack on a positively charged carbon of the substrate. This leads to the formation of a tetrahedral intermediate of limited stability. Final proton transfer from the proton shuttle residue (His dyad in the case of HDAC6), where the proton has been deposited during the first half-reaction, to the substrate (typically the nitrogen of a peptide/amide bond, here to the N_3_ nitrogen of the oxadiazole ring) leads to cleavage of the C–O (in peptides C–N) bond and opening of the ring. After a certain repositioning of the substrate that has been shown to be associated with a free energy cost of ∼12 kcal·mol^–1^, the cleaved oxadiazole ring adopts an almost identical conformation in the active site as the natural substrate—an *N*-Ac-Lys residue. It thus appears that the second hydrolytic step is close to the “native” HDAC6 activity (*k*_cat_ ≈ 1 s^–1^).^37^

Besides testing some variants of the computational protocol (see below), we also carried out computations where the CF_2_H group adjacent to the oxadiazole ring was replaced with CH_3_ (to obtain compound **10**). Note that we have not carried out full QM/MM optimizations of all HDAC6/**10** intermediates but performed only single-point energy calculations of the final structure by substituting two hydrogens for two fluorines to quickly assess the effect of the substitution. We may conclude that calculations fully support the experimental observation that a CF_2_H or CF_3_ group is a prerequisite for the oxadiazole ring cleavage to occur. The computed values (without entropy corrections) are 0.0 (starting at **MC**_**S1**_); 9.3; 4.3; 28.2; −11.4 kcal·mol^–1^ (ending at **P**_**S1**_) for the first hydrolytic step (cf. Figure 10); and 0.0 (set to 0 for **MC**_**S2**_′); 8.7; 7.4; 18.3; 3.8 kcal·mol^–1^ (**P**_**S2**_) for the second hydrolytic step. It can be concluded that (approximate) activation energies along the reaction pathway are for all elementary steps 2–8 kcal·mol^–1^ higher than for the “parent” compound **8**. This seems to be ample numerical evidence explaining the observed (and anticipated) non-reactivity of **10**, admitting that the computed differences may rather represent upper limits to ΔΔ*G*′_comp_ (CF_2_H/CH_3_).

We have also looked for simple descriptors which would allow us to explain the reactivity difference in the **7**–**10** series (evaluated numerically in the previous paragraph). We may remind that it was observed (Table 1) that at least monofluorination of the methyl group is necessary for the oxadiazole warhead to be active. The highest barriers in both hydrolytic stages are associated with the cleavage of the C–O bonds in tetrahedral intermediates (**TI**_**S1**_, **TI2**_**S2**_, and the corresponding transition states, **TS2**_**S1**_ and **TS2**_**S2**_). These transition states feature negatively charged species coordinated to the zinc(II), unlike “starting states” **MC**_**S1**_ and **MC**_**S2**_′, which feature a neutral species. The negative charge is, in both cases, localized on the β atoms relative to the CF_2_H group. This is a situation similar to the deprotonation of fluoroacetic acids, and we can thus compare the Gibbs free energy change between these processes. A value for ΔΔ*G*_CF_2_HCOOH/CH_3_COOH_ of (4.76–1.34) × 1.36 kcal mol^–1^ = 4.7 kcal mol^–1^ lies in the middle of the 2–8 kcal mol^–1^ “destabilization” range of the charged intermediates.^36^ This destabilization applies to all species that carry a negative charge on the nitrogen/oxygen connected to the β carbon atom in the (difluoro)acetate moiety.

Finally, we wanted to check the robustness of the calculated values with respect to the variation of a few variables in a computational protocol (change of the functional, solvation method, and presence/absence of entropic corrections). These are summarized in Table 2.

**Table 2:** Computed Energy Profiles Employing Various Computational Protocols

system	Δ*G*_comp_′ (TPSS)^b^	Δ*G*_comp_′ (B3LYP)^c^	Δ*G*_comp_′ (BP86/CRS)^d^	Δ*G*_comp_′ (BP86/CRS)_noWat^e^	Δ*E*_comp_′ (TPSS)^f^
**MC**_**S1**_	**0.0**^a^	0.0	0.0	0.0	0.0
**TS1**_**S1**_	**4.4**	8.5	4.8	6.0	5.1
**TI**_**S1**_	**–2.7**	–0.6	–2.9	–3.1	–4.0
**TS2**_**S1**_	**18.3**	21.0	19.4	15.5	20.7
**P**_**S1**_	**–15.0**	–18.3	–14.4	–15.1	–16.8
**MC**_**S2**_^**gm**^	**–16.0**	–15.7	–16.8	–13.0	–16.0
**MC**_**S2**_′	**–3.7**	–3.7	–3.7	–3.7	–1.4
**TS1**_**S2**_	**2.2**	4.5	4.3	4.4	4.6
**TI**_**S2**_	**–3.4**	–2.5	–2.1	–3.6	–3.3
**TS2**_**S2**_	**5.9**	9.3	5.2	5.3	10.7
**P**_**S2**_	**–8.3**	–6.7	–9.8	–6.9	–7.0
**P**_**FIN**_	**0.0**	0.0	0.0	0.0	0.0

a All values are in kcal·mol^–1^.

b *G*_comp_′(TPSS) = *E*(QM(TPSS-D3/def2-TZVP)/COSMO(ε_r_ = 8))//QM/MM_geometry_ + *E*_ZPVE_ + *RT* ln *Q* + *RT*, as reported above.

c *G*_comp_′(B3LYP) = *E*(QM(B3LYP-D3/def2-TZVP)/COSMO(ε_r_ = 8))//QM/MM_geometry_ + *E*_ZPVE_ + *RT *ln *Q* + *RT*.

d *G*_comp_′(BP86/CRS) = *E*(QM(BP86-D3/def2-TZVPD)/COSMO-RS(1-octanol))//QM/MM_geometry_ + *E*_ZPVE_ + *RT* ln* Q* + *RT*.

e *G*_comp_′(BP86/CRS)_noWat; defined as Δ*G*_comp_′(BP86/CRS), but 14 explicit water molecules removed from the QM and treated implicitly.

f *E*_comp_′(TPSS) = *E*(QM(TPSS-D3/def2-TZVP)/COSMO(ε_r_ = 8))//QM/MM_geometry_; without entropy corrections.

It can be seen that the values computed by various computational protocols do not differ significantly. Exchanging the TPSS-D3 functional for B3LYP-D3 (the probably most frequently used functional in contemporary quantum chemistry, perhaps very slightly overestimating activation energies for hydrolysis by zinc(II)-hydrolytic enzymes)^21^ does lead to marginally higher activation energies, but the RDS (**TI**_**S1**_**→ TS2**_**S1**_) activation energy remains almost the same (21.0 vs. 21.6 kcal·mol^–1^). Both values are in very good agreement with the estimate of the rate constant for the conversion of the oxadiazole in the order of 10^–3^ s^–1^ (Figure 3). What is even more striking, in our opinion, is the agreement between the standard PCM (COSMO) protocol and an advanced COSMO-RS protocol, which employs a completely different philosophy for computations of solvation-free energies. It can be noted that the BP86 functional is a prerequisite for the COSMO-RS protocol. Furthermore, the (free) energies along the reaction pathway do not vary significantly after the removal of 14 water molecules that were present in the QM system in the QM/MM (and QM) calculations, cf. Δ*G*_comp_′(BP86/CRS) vs. Δ*G*_comp_′(BP86/CRS)_ noWat values. Finally, the last column shows the Δ*E*_comp_′(TPSS) values, which differ from the values reported throughout this work by the absence of the thermal energy and entropic terms (*E*_ZPVE_ + *RT* ln *Q* + *RT*). All in all, we consider the computed energy profiles as quite stable with respect to various flavors of the computational protocol, which gives us confidence in the conclusions presented herein.

## Conclusions

By combining X-ray crystallography and enzyme kinetics *in vitro* and in cells with QM/MM and QM calculations, we have elucidated the complete reaction mechanism for the conversion of oxadiazole inhibitors by HDAC6 to their respective hydrazides. Our detailed mechanistic analysis may open new avenues in the creation of next-generation inhibitors for this highly important biopharmaceutical target possessing increased specificity and potency. Accordingly, efforts are currently underway to design, synthesize, and test structurally related inhibitors that may be less prone to hydrolytic ring opening and thus afford more stable HDAC6-selective inhibitors of clinical potential.

## Materials and Methods

If not stated otherwise, reagents were purchased from Sigma-Aldrich.

### Expression and Purification of Human HDACs 1–11

Large-scale expression of recombinant human HDACs was carried out as described previously.^32,37^ Briefly, HEK293/T17 cells were transiently transfected with expression pMM222 plasmids comprising N-terminally tagged (TwinStrep-FLAG-HALO tag; Figure S1) HDAC genes using linear poly(ethyleneimine) (PEI, Polysciences Inc.). Cells were harvested three days post-transfection, and cell pellets were resuspended in the lysis buffer (100 mM Tris-HCl, 10 mM NaCl, 5 mM KCl, 2 mM MgCl_2_, and 10% glycerol at pH 8.0). Cells were disrupted by sonication, cell lysates were cleared by centrifugation, and soluble fusion proteins were first purified by Streptactin affinity chromatography (IBA). For all HDACs but HDACs 1, 10, and 11, the N-terminal tag was cleaved off overnight by the addition of TEV protease (10:1 HDAC/TEV ratio). Size-exclusion chromatography on a Superose 6 column (GE Healthcare Bio-Sciences; running buffer 30 mM HEPES, 140 mM NaCl, 10 mM KCl, 3% glycerol, and 0.25 mM TCEP) was used as the final purification step for all HDAC constructs.

### Expression and Purification of zHDAC6 (440–798)

The second catalytic domain of *Danio rerio* HDAC6 (amino acids 440–798; zHDAC6) was expressed in RIPL *Escherichia coli* as described previously using the pEC566-zHDAC6 expression vector.^16,38^ Briefly, bacteria were incubated at 37 °C with shaking (250 rpm) until the optical density reached an OD_600_ = 0.5. Then, the culture was cooled to 18 °C for 1 h, and recombinant protein expression was induced by the addition of 75 μM isopropyl β-d-1-thiogalactopyranoside together with 200 μM ZnSO_4_. The culture was grown at 18 °C for an additional 18 h and harvested by centrifugation at 5500*g* for 15 min. The cell pellet was resuspended in the lysis buffer (50 mM phosphate, 300 mM NaCl, 10% glycerol, and 20 mM imidazole at pH 8; 5 mL g^–1^ bacterial pellet) and lysed by three passages through EmulsiFlex (Avestin). Cell lysates were centrifuged (15,000*g* at 4 °C for 20 min), and the fusion protein in the supernatant was purified *via* Ni-NTA chromatography. Fractions containing the His-MBP-HDAC6 fusion were concentrated to 2 mg mL^–1^ and cleaved overnight by the addition of TEV protease (zHDAC6/TEV 1:20 ratio). The cleaved tag and the TEV protease were sequentially removed using amylose (New England Biolabs) and HisTrap columns (Cytiva), and zHDAC6 was further purified by size-exclusion chromatography on a HiLoad Superdex 75 pg column (GE Healthcare Bio-Sciences). Fractions containing zHDAC6 were pooled, concentrated to 10 mg mL^–1^, flash-frozen in liquid nitrogen, and stored at −80 °C. The purity of protein preparations was monitored by SDS-PAGE (Figure S2).

### Site-Directed Mutagenesis

The zHDAC6 mutants (Y745F and H574A) were constructed using the Quick-change site-directed mutagenesis protocol with the pEC566-zHDAC6 expression plasmid as a template. Mutagenic primer sequences are shown in Table S2. Both mutants were expressed and purified as described above for wild-type zHDAC6 (Figure S2).

### IC_50_ Determination

Optimized fluorometric assays were used to determine inhibition constants of studied compounds against individual HDAC isoforms as described previously.^32^ Briefly, reactions were performed in the activity buffer comprising 50 mM HEPES, 140 mM NaCl, and 10 mM KCl at pH 7.4 supplemented with 1 mg mL^–1^ bovine serum albumin (BSA), 1 mM tris(2-carboxyethyl)phosphine (TCEP), 10 μM substrates, and optimized concentrations of HDACs. The Ac-Gly-Ala-[Lys-Ac]-7-amino-methylcoumarylamide (AMC) substrate was used to profile HDACs 1, 2, 3, and 6; Boc-[Lys-TFA]-7-amino-methylcoumarylamide was used for HDACs 4, 5, 7, 8, and 9 (both substrates: Bachem); fluorescein-labeled *N*8-acetylspermidine was used for HDAC10;^32^ and an internally quenched TNFα-derived peptide derivative was used for HDAC11.^39^ Deacetylation reactions were monitored either by high-performance liquid chromatography (HPLC) (HDAC10) or by quantification of released AMC (λ_ex_/λ_em_ = 365/440 nm) using a CLARIOstar plate reader (BMG Labtech GmbH). For IC_50_ determination, HDACs were preincubated with dilution series of a tested inhibitor for 15 min prior to substrate addition, and inhibition data were fitted in the GraphPad Prism software (GraphPad Software) using nonlinear regression analysis.

### HEK293T Cells Stably Expressing Human HDAC6

HEK293 cells stably transduced with full-length human HDAC6 were generated following the protocol published previously.^40^ Briefly, Lenti-X 293T and HEK293T cells were grown in T75 flasks in the DMEM/F-12/10% fetal bovine serum (FBS) medium under an atmosphere containing 5% CO_2_ at 37 °C. The Lenti-X 293T cells were transfected with the combination of pMD.2G, psPAX2, and pHR_CMV_MM320_hsHD6 (Figure S2) using Lipofectamine2000 (Invitrogen) according to the manufacturer’s protocol. Three days post-transfection, the virion-rich medium was collected by centrifugation, supplemented with polybrene (final concentration 10 μg mL^–1^), and added to adherent HEK293T cells grown to 100% confluency. Three days post-infection, transduced HEK293T cells were expanded in FreeStyle 293 medium (GIBCO) supplemented with 1% FBS in an Erlenmeyer flask (suspension culture, 110 RPM). Transduction efficacy was determined by flow cytometry analysis to be >95%.

### LC–MS/MS Quantification

Mass spectrometry quantification of inhibitors/reaction products was carried out with a Shimadzu LCMS-8040 instrument (Shimadzu, HPLC Prominence system). Analytes were separated on a Kinetex XB-C18 2.6 μm, 100 Å column (50 × 2.1 mm; Phenomenex) in a linear 15–60% CH_3_CN/H_2_O gradient containing 0.1% formic acid at a flow rate of 0.8 mL min^–1^ over 4 min. Multiple reaction monitoring (MRM) parameters were optimized for each analyte, and corresponding major CID fragments were used for quantification. Analytes were quantified using corresponding calibration curves generated from pure compounds dissolved in water.

### Inhibitor Hydrolysis *In Vitro*

Twenty micromolar solutions of compounds were mixed with 1 μM zHDAC6 in a 96-well plate in an assay buffer comprising 50 mM HEPES, 140 mM NaCl, and 10 mM KCl at pH 7.4. The sealed plate was placed into the autosampler of the Shimadzu LCMS-8040 system with the temperature control set to 25 °C. Four microliter aliquots of reaction mixtures were injected into the system directly from the 96-well plate, and inhibitors/reaction products were quantitated as described above.

### Inhibitor Hydrolysis Cell Lysates

HEK293T cells and the HEK293T cell line stably transfected with human HDAC6 were grown in FreeStyle 293 medium supplemented with 1% FBS. Cells were harvested by centrifugation at 500*g* for 5 min, and cell pellets were resuspended in the lysis buffer (50 mM HEPES, 140 mM NaCl, 10 mM KCl, and 0.5% NP-40 at pH 7.4). Following short sonication (3 × 5 s), the cell lysates were centrifuged at 20,000*g* for 10 min, supernatants were transferred to fresh tubes, and the total protein concentration was adjusted to 4 mg mL^–1^ by the addition of the lysis buffer. Tested compounds were mixed with cell lysates at final compound and protein concentrations of 20 μM and 2.5 mg mL^–1^, respectively, and incubated at 37 °C. At defined time intervals, 40 μL of reaction aliquots were removed and the reaction was stopped by the addition of 120 μL of ice-cold acetonitrile + 0.1% acetic acid. Following 15 min incubation on ice, stopped reactions were centrifuged at 20,000*g* for 10 min, and supernatants were analyzed by LC–MS as described above.

### Crystallization and Data Collection

For co-crystallization of the HDAC6/**8** complex, a 0.12 μL drop of the protein solution [10 mg mL^–1^ zHDAC6 440–798, 50 mM HEPES pH 7.5, 100 mM KCl, 1 mM TCEP, 5% glycerol (v/v), 4 mM compound **8**, and 5% DMSO] was mixed with 0.1 μL of the reservoir solution containing 17% PEG 3350, 0.2M KSCN (Hampton Research), and 0.1M Bis-Tris at pH 6.8. Crystals were grown by the hanging drop vapor diffusion method at 283.15 K and appeared after several days. To overcome the low success rate of crystallization, strike seeding was performed. The seed stock was prepared by crushing zHDAC6 crystals obtained under identical conditions previously. Crystals were vitrified in liquid nitrogen in the mother liquor supplemented with 15% glycerol. The diffraction data were collected at 100 K using a METALJET liquid metal X-ray source D8 VENTURE (Excillium) in The Center of molecular structure, Vestec, Czech Republic, at an X-ray wavelength of 1.34 Å. The complete dataset was collected from a single crystal using the Bruker PHOTON II detector (Bruker, Table S3).

### Structure Determination and Refinement

The difference Fourier method was used to determine the structure of the zHDAC6/**4** complex using the zHDAC6-CD2/Tubastatin A complex (PDB ID: 6THV) as a starting model.^38^ Refinement was performed using REFMAC5.8,^41^ and manual editing was done in COOT.^42^ Ligand topology and coordinates were generated with AceDRG,^43^ and the inhibitor was fitted into the |*F*_o_| – |*F*_c_| electron density map in the final stages of the refinement. Approximately 5% of randomly selected reflections were utilized as an *R*_free_ set. The final model was validated using the MolProbity server.^44^ The data collection and structure refinement statistics are summarized in Table S3. Atomic coordinates and corresponding structure factors for the zHDAC6/**4** complex have been deposited at the Protein Data Bank (PDB) as the 8BJK entry.

### Computational Details

#### Protein Setup and Equilibration

We employed similar computational protocols and an analogous QM/MM setup as those previously successfully applied in mechanistic and structural studies of various metalloenzymes,^45−47^ including an experimentally calibrated QM/MM study of the dizinc hydrolytic enzyme, glutamate carboxypeptidase II (GCPII).^22^ GCPII shares many structural and mechanistic features with HDAC6 studied herein: activation of a water molecule for nucleophilic attack on the carbon atom of the substrate, formation of the tetrahedral intermediate, and proton-assisted cleavage of the C–N (or in the case of the first hydrolysis of oxadiazole, C–O) bond.

The initial protein structure was prepared from the experimental X-ray structure of the [HDAC6+**4**] complex reported herein. The hydrazide moiety was completed to a full oxadiazole ring, roughly to a position defined in ref (24). Next, the initial X-ray structure was fully equilibrated employing standard protocols: (a1) minimizing the positions of all hydrogen atoms added to the initial crystal structure corresponding to the standard protonation states of amino acid side chains at pH = 7, which correlates well with the pH = 6.8–7.4 at which the X-ray structure was determined (for the histidine residues, the protonation status was assigned based on a careful inspection of the hydrogen-bond network around the residue and the solvent accessibility; i.e., histidines 503, 573, 574, and 683 were assumed to be protonated on the Nδ atom; histidines 455, 462, 463, 487, 507, 516, 614, 615, 635, 689, 724, 727, and 790 on the Nε atom; and histidines 456, 477, and 621 on both, thus being in the His^+^ protonation state); (b1) running a 1 ns simulated annealing molecular dynamics (MD) computation (see below for technical details), followed by the final minimization of the whole system (with all non-hydrogen atoms kept at their crystallographic positions throughout both steps).

Next, a solvation sphere with a radius of 36 Å (3472 of TIP3P water molecules in total) was added. The same equilibration procedure was then repeated: (a2) minimizing the positions of all hydrogen atoms and all added waters (including their oxygen atoms); (b2) running a 1 ns simulated annealing molecular dynamics computation, followed by the final minimization of the whole system, all with the same set of atoms fixed as in (a2). In (a2), a “CAP procedure” in the Amber program,^48^ which consists of a centric force applied to the water molecules in the solvation sphere was used to ensure that these water molecules do not dissociate from the system during higher-temperature MD annealing.

#### Molecular Dynamics Calculations: Technical Details of MD Annealing

The same standard MD annealing protocol has been used as in the previous QM/MM studies.^45−47^ Within the 180 ps MD trajectory, the system was heated to 353 K and cooled slowly down to 0 K, which is considered to be sufficient for the equilibration, since heavy atoms from the protein are fixed. The bond lengths involving hydrogen atoms were not constrained. Electrostatic interactions were treated using the particle-mesh Ewald method with a real-space cut-off of 15 Å. Temperature control was accomplished using the Berendsen weak-coupling algorithm with coupling constants of 0.05–1 ps. The MD time step was 1 fs, and the non-bonded pair list was updated every 50 fs. All MD simulations were performed with explicit water molecules using the TIP3P water model.

#### Definition of the Quantum System in QM/MM Calculations

The quantum system comprised 317 atoms and included side chains (*s*) or backbone/main chain atoms (*m*), or whole residues (*w*) of the following amino acid residues: H462(*m*_N_)-H463(*w*)-P464(*w*)-E465(*m*_C_), S531(*s*), H573(*s*), H574(*s*), C581(*m*_N_)-G582(*w*)-F583(*w*)-C584(*w*)-F585(*m*_C_), D612(*s*), H614(*s*), F643(*s*), D705(*s*), L712(*s*), L741(*m*_N_)-E742(*w*)-G743(*w*)-G744(*w*)-Y745(*w*)-N746(*m*_C_), Zn^2+^ ion, inhibitor/substrate, and 13 water molecules. Here, the AA_*i*_(*m*/*w*/*s*)-···-AA_*i*+*k*_ notation denotes the continuous chain in the protein, whereas *m*_N_ (H_J_-C(=O)- unit) and *m*_C_ (NH-CHH_J_H_J_′ unit) are the N- and C-terminal QM/MM caps of the chains (H_J_ is the junction atom, hydrogen in QM and MM1, and carbon in the MM3 calculation, see below). The part of the protein that was allowed to relax in the QM/MM calculations comprised an additional 44 residues and 42 water molecules in the vicinity of the QM system (i.e., any residue/water with an atom with *R* < 2.5 Å from any atom of the QM region was included as a whole).

#### QM/MM and QM Calculations

The *ComQum* software was used for all QM/MM calculations. A detailed description of the contributions to the total QM/MM energy is summarized in the Computational Details section in the Supporting Information, while more technical details can be found in refs (49−52). In brief, the *ComQum* package combines *Turbomole* (v. 6.6)^53^ and *Amber* programs to carry out the QM and MM calculations, respectively. It employs electrostatic embedding, the hydrogen link-atom scheme, and a microiterative approach,^50^ i.e., the MM system is fully relaxed after each optimization step in the QM region. The total QM/MM energy is then calculated as:
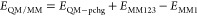
1where *E*_QM-pchg_ corresponds to the QM energy of System 1 embedded in a set of point charges of Systems 2 and 3 (MM part; the self-interaction of point charges not included in the *E*_QM/MM_ term), *E*_MM123_ is the MM energy of the entire system (MM charges of the System 1 zeroed), and *E*_MM1_ is the MM energy of System 1, again with the MM charges of the System 1 zeroed. Note that the MM part thus formally consists of Systems 2 (MM part allowed to relax) and 3, which are (energetically) treated on the same footing. However, System 3 atoms (the outer part of the protein) are fixed at their original crystallographic positions.

In the QM part of the QM/MM calculations, geometry optimizations were carried out using the BP86 functional^54^ with the DGauss-DZVP basis set,^55^ including the empirical zero-damping dispersion correction (D3),^56^ and expedited by the RI-J approximation.^57^ The MM calculations were performed employing the Amber ff14SB force field.^58^ The transition states were obtained as one- or two-dimensional scans of QM/MM potential energy surfaces (PESs). To obtain the first transition state and tetrahedral intermediate in each of the two hydrolytic step structures, 1D scan along the O···C reaction coordinate (where O is the oxygen of the Zn-bound hydroxide nucleophile, and C is on the oxadiazole ring) seemed to be sufficient. For the second transition state and to reach the final product, the variation of the C–N (C–O in the first hydrolytic step) bond distance was coupled with the hydrogen atom transfer from the H574 serving as the proton shuttle to the emerging amine functional group.

For the QM(BP86-D3/DGauss-DZVP/MM) equilibrium geometries, the single-point energies were evaluated with BP86-D3,^54^ TPSS-D3,^59^ and B3LYP-D3^25^ methods and the def2-TZVP basis set^60^ embedded in the homogeneous environment represented by the conductor-like screening model (COSMO)^61^ with a dielectric constant ε = 8 and optimized radii used for the standard elements (2.0 Å for Zn ion). This approach has been recently shown to yield fairly accurate and stable molecular energies for metalloenzymatic reactions.^45−47^

#### COSMO-RS and Entropic Corrections

In addition, we have also employed a conductor-like screening model for realistic solvation, the COSMO-RS method,^62,63^ as implemented in the COSMOtherm21 program (Dassault Systèmes). The COSMO-RS method was used in conjunction with the “BP_TZVPD_FINE_21.ctd” parametrization file and FINE cavities ($cosmo_isorad keyword). The quantum system was “embedded” in 1-octanol (representing the “average” permittivity of a protein), whereas H_2_O (to assess the free energy of binding in connecting the first and second hydrolytic steps) and CHF_2_COO^-^ were solvated in water. The final free energies of the species involved were obtained via the following formula:
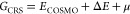
2where *E*_COSMO_ is BP86-D3/COSMO (ε = ∞) energy of the conformer, Δ*E* is the averaged correction for the dielectric energy, and μ is the chemical potential of the conformer. It can be mentioned that throughout our previous studies,^28^ we observed that the COSMO-RS method slightly, by ∼3 kcal·mol^–1^ in free energy, (artificially) favors the dissociation of water molecules from the “cluster” (of water molecules, or enzyme active site, etc.). This empirical correction was applied for the water dissociation free energy computed herein (**P**_**S1**_ → **MC**_**S2**_^**gm**^ process).

The zero-point vibrational energy, thermal corrections to the Gibbs free energy, and the entropic terms were calculated by employing standard formulas of statistical thermodynamics,
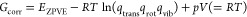
where *E*_ZPVE_ is the zero-point vibrational energy, whereas *RT* ln(*q*_trans_*q*_rot_*q*_vib_) is the entropic term obtained from the rigid-rotor/harmonic oscillator (RRHO) approximation in which a free rotor model was applied for low-lying vibrational modes under 100 cm^–1^ with a smoothing function applied (sometimes denoted as quasi-RRHO, or RRFRHO approximation), employing Grimme′s thermo program.^64^ The computation of vibrational frequencies was done in two ways, always employing the COSMO (ε_r_ = 8) solvation model and numerical calculations of frequencies. In the first and in our opinion more rigorous approach, the QM system was truncated to a minimum core, approx. ∼100 atoms (structure Small_Model.xyz deposited in the SI), and was freely geometry-optimized without any constraints. The final equilibrium geometries preserved essential structural characteristics of the original QM/MM equilibrium geometries and possessed only very few imaginary frequencies (in addition to the imaginary frequency corresponding to the “reactive” mode in the transition states) that are essentially numerical noise. These are flipped into positive values—an approach that has been previously well-tested and leads only to negligible errors.^65−67^ We also tried to compute numerical frequencies for the full-size QM system, keeping the junction atoms fixed in geometry reoptimization of the QM system and then projecting them out via the *FreezeNuclei* option in frequency calculation (by assigning them infinite mass). Surprisingly, the computed *G*_corr_, albeit numerically less stable (i.e., more imaginary frequencies), mostly agreed, to within 1 kcal·mol^–1^, with the approach 1. In three out of twelve cases, where the disagreement was slightly greater, it was obvious that the problems were in the numerical instability of the larger QM model, i.e., approach 2. Therefore, we corrected the free energy values by using *G*_corr_ from approach 1, but the agreement gives us certain confidence in evaluating the entropy of the discussed contributions. Finally, in addition/dissociation of species (H_2_O or CHF_2_COO^–^), we employed the 1.9 Δ*n* kcal·mol^–1^ “standard state” conversion, where Δ*n* is the molar change in the process. It must be re-emphasized that all of the above computational approaches have been tested and verified against experimental data in our previous studies.^22,46,68−70^

### Chemical Synthesis

All solvents used for synthesis were obtained from commercial sources. All chemicals were purchased from Sigma-Aldrich, TCI, or Combi-Blocks and were used without further purification. Thin-layer chromatography (TLC) was performed on silica gel 60 F_254_-coated aluminum sheets (Merck). Products were purified by preparative scale HPLC on a JASCO PU-975 instrument (flow rate 10 mL min^–1^) equipped with a UV-975 UV detector and a Waters YMCPACK ODS-AM C_18_ preparative column (5 μm, 20 × 250 mm). The purity of compounds was assessed on an analytical JASCO PU-1580 HPLC (flow rate 1 mL min^–1^, invariable gradient from 2 to 100% acetonitrile in water in 30 min) with a Watrex C_18_ analytical column (5 μm, 250 × 5 mm). ^1^H and ^13^C NMR spectra were measured using Bruker AVANCE III HD 400 MHz, Bruker AVANCE III HD 500 MHz, and Bruker AVANCE III 600 MHz instruments. The internal signal of TMS (δ 0.0, CDCl_3_) or the residual signals of CDCl_3_ (δ 7.26) or CD_3_OD (δ 3.31) were used for standardization of ^1^H NMR spectra. For ^13^C NMR spectra, the residual signals of CDCl_3_ (δ 77.16) or CD_3_OD (δ 49.00) were used. NMR spectra were recorded at room temperature unless noted otherwise. Chemical shifts are given in the δ scale; coupling constants (*J*) are given in Hz. The ESI mass spectra were recorded using a Micromass ZQ mass spectrometer (Waters) equipped with an ESCi multimode ion source and controlled by MassLynx software. Low-resolution ESI mass spectra were recorded using a quadrupole orthogonal acceleration time-of-flight tandem mass spectrometer (Q-Tof micro, Waters) and high-resolution ESI mass spectra using a hybrid FT mass spectrometer combining a linear ion trap MS and Orbitrap mass analyzer (LTQ Orbitrap XL, Thermo Fisher Scientific). The conditions were optimized for suitable ionization in the ESI Orbitrap source (sheath gas flow rate of 35 au, auxiliary gas flow rate of 10 au of nitrogen, source voltage of 4.3 kV, capillary voltage of 40 V, capillary temperature of 275 °C, tube lens voltage of 155 V). The samples were dissolved in methanol and applied by direct injection. Analytical data are provided in the Supporting Information.

## Supporting Information Available

Synthetic details and compound characterization, Tables S1–S3, Figures S1, S2 (PDF)
^1^H NMR (401 MHz, CD_3_OD), ^13^C NMR (101 MHz, CD_3_OD), ESI MS, HR ESI MS (PDF)
Compound characterization checklist (XLSX)
Equilibrium geometries of all studied systems (QM regions in QM/MM simulations and one full PDB model) (ZIP)

## Accession Codes

Atomic coordinates and corresponding structure factors for the zHDAC6/**4** complex have been deposited at the Protein Data Bank (PDB) as the 8BJK entry. Authors will release the atomic coordinates upon article publication.
