# Supplementary material for: Comprehensive Mechanistic View of the Hydrolysis of Oxadiazole-Based Inhibitors by Histone Deacetylase 6 (HDAC6)
Source: ACS Chem Biol. 2023 Jul 3;18(7):1594–610. doi: 10.1021/acschembio.3c00212 (PMC10367051; doi:10.1021/acschembio.3c00212)
Supplement: Supplementary file 1 — cb3c00212_si_001.pdf [file cb3c00212_si_001.pdf]

SUPPORTING INFORMATION

Comprehensive Mechanistic View of the Hydrolysis of Oxadiazole-Based Inhibitors by Histone Deacetylase 6 (HDAC6)

Lucia Motlová[1], Ivan Šnajdr[2], Zsófia Kutil[1], Erik Andris[2], Jakub Ptáček[1], Adéla Novotná[2], Zora Nováková[1], Barbora Havlínová[1], Werner Tueckmantel[3], Helena Dráberová[1], Pavel Majer[2], Mike Schutkowski[4], Alan Kozikowski[3], Lubomír Rulíšek[2], Cyril Bařinka[1]**

[1]Institute of Biotechnology of the Czech Academy of Sciences, BIOCEV, Prumyslova 595, 252 50 Vestec, Czech Republic

[2]Institute of Organic Chemistry and Biochemistry of the Czech Academy of Sciences, Flemingovo náměstí 2, 166 10, Prague 6, Czech Republic

[3]StarWise Therapeutics LLC, University Research Park, Inc., Madison, WI 53719, United States

[4]Department of Enzymology, Charles Tanford Protein Center, Institute of Biochemistry and Biotechnology, Martin-Luther-University Halle-Wittenberg, 06120 Halle, Germany

Corresponding authors:

cyril.barinka@ibt.cas.cz; rulisek@uochb.cas.cz

Supporting Information Contents

Supplementary Tables S1-S3 ....p. S3-S6

Supplementary Figures S1, S2 ....p. S7-S8

Analytical Data of the Synthesized Compounds ....p. S9-S16

References...p. S16

Geometry coordinates of the optimized structures and primary energetic data as discussed in the main text are provided in a separate archive.

Table S1: The most important *alternative* pathways studied computationally.

State	Alternative(s): Structural Description	Outcome of the Calculation(s)	SI Structure Filename
**MC$_{S1}$**	Alt1: started with oxadiazole ring rotated 90 degrees to allow for a potential flip of the ring to align with nitrogens in the hydrazide structure and catalytic water attacking from the other side of the ring	Rotates back to the **MC$_{S1}$** arrangement of the oxadiazole ring, but water stays on the other side; energy is ~20 kcal.mol^{-1} higher than **MC$_{S1}$**	MC_S1_Alt1.xyz
	Alt2: started with oxadiazole ring rotated 90 degrees and yet another different position of catalytic water	Converges almost exactly to **MC$_{S1}$**	*n.a.* (the same as **MC$_{S1}$** deposited in the SI)
**TS1$_{S1}$**	Trajectory well defined as 1-D scan of the $R(C_{oxadizole}\ldots O_{OH-})$ distance	Structures with $R(C_{oxadizole}\ldots O_{OH-})$ = 1.7-1.9 Å almost isoenergetic to within 0.2 kcal.mol^{-1} (= very flat PES around **TS1$_{S1}$**)	n.a. (almost identical to **TS1$_{S1}$** deposited in the SI)
**TI$_{S1}$**	Alt1: oxadiazole ring rotated by 45 degrees toward His dyad *before* the proton is transferred	Restrained optimization ended up in the structure that was ~10 kcal.mol^{-1} higher in energy	TI_S1_Alt1.xyz
**TS2$_{S1}$**	S_24_22: 2-D scan of cleaved C-O distance (in this particular case 2.4 Å) and $N_4\ldots H_{His+}$ distance (2.2 Å)	QM/MM optimization provided almost isoenergetic structures to the reported **TS2$_{S1}$**	TS2_S1_24_22.xyz
	S_24_21: 2-D scan of cleaved C-O distance (in this particular case 2.4 Å) and $N_4\ldots H_{His+}$ distance (2.1 Å)	QM/MM optimization provided almost isoenergetic structures to the reported **TS2$_{S1}$**	TS2_S1_24_21.xyz
	S_h21_min: very shallow minimum at around $R(N_4\ldots H_{His+})$ = 2.0 Å	Already ~ 4 kcal.mol-1 lower than the **TS2$_{S1}$**	TS2_S1_h21_min.xyz
	S_h19_min: unconstrained optimization starting from the point $R(N_4\ldots H_{His+})$ = 1.9 Å	Collection of minimizations steps where the breaking of the C-O bond and rotation of the (formerly) oxadiazole ring occurs	TS2_S1_movie_min.xyz
		*In summary, all efforts to find better **TS2$_{S1}$** candidate provided at best the isoenergetic*	

		structures with the $\boldsymbol{TS2_{S1}}$ *reported throughout this work*	
$\mathbf{P_{S1}}$	Alt1: Structure where **6** is bound via carbonyl oxygen, only N_3 protonated	Approx. 15 kcal.mol-1 higher than $\mathbf{P_{S1}}$ reported in the work	P_S1_Alt1.xyz
	Alt2: Structure where **6** is bound via carbonyl oxygen, both N_3 and N_4 protonated	Very high in energy	P_S1_Alt2.xyz
	Alt3: Structure where **6** is bound via carbonyl oxygen, both N_3 and N_4 protonated, different orientation	Very high in energy	P_S1_Alt3.xyz
	Alt4: Structure where **6** is bound via carbonyl oxygen, both N_3 and N_4 protonated, different orientation	~5 $kcal.mol^{-1}$ lower in energy than Alt1, but still higher than $\mathbf{P_{S1}}$	P_S1_Alt4.xyz
$\mathbf{MC_{S2}}$	Alt1: coordination of **6** by the N_3 nitrogen, added water in the second solvation sphere	Approx. 7 $kcal.mol^{-1}$ higher in energy than $\mathbf{MC_{S2}^{gm}}$	MC_S2_Alt1.xyz
	Alt2: bidentate coordination of **6** by the N_3 nitrogen and carbonyl oxygen, added water in the first solvation sphere, pentacoordination, one Asp de-coordinated	Approx. 9 $kcal.mol^{-1}$ higher in energy than $\mathbf{MC_{S2}^{gm}}$	MC_S2_Alt2.xyz
$\mathbf{TS1_{S2}}$	Trajectory well defined as 1-D scan of the $R(C_{cpd6}…O_{OH-})$ distance	Structures with $R(C_{cpd6}…O_{OH-})$ = 1.8-2.0 Å almost isoenergetic to within 1 $kcal.mol^{-1}$ (= very flat PES around $\mathbf{TS1_{S1}}$)	n.a. (almost identical to $\mathbf{TS1_{S2}}$ deposited in the SI)
$\mathbf{TI_{S2}}$	Only one structure $\mathbf{TI_{S2}}$ obtained		n.a.
$\mathbf{TS2_{S2}}$	Direct proton unassisted C-N bond cleavage	Leads to structure high in energy (> 20 $kcal.mol^{-1}$) already at $R(C_{cpd6}…O_{OH-})$ = 2.2 Å	TS2_S2_Alt1.xyz
	Hypothetical proton transfer from Y745 to N_3 of oxadiazole	Unfeasible, goes uphill without any chemistry occuring, Y745 cannot serve as proton shuttle	TS2_S2_Alt2.xyz
	Internal proton transfer from the OH of the tetrahedral intermediate to the N_3	Unfeasible, goes uphill (> 20 $kcal.mol^{-1}$) without any chemistry occuring	TS2_S2_Alt3.xyz
$\mathbf{P_{S2}}$	Alt1: Cleaved Acetate Bound as a Ligand to Zn(II), hydrazide decoordinated	Almost isoenergetic with $\mathbf{P_{S2}}$, the barrier of its conversion to the $\mathbf{P_{S2}}$ around 10 $kcal.mol^{-1}$	P_S2_Alt1.xyz

Table S2: Mutagenic primes for constructing zHDAC6 mutants.

zHD6_Y745F_F	CTGGAGGGTGGTTTTAACCTCACTTCCATC
zHD6_Y745F_R	GATGGAAGTGAGGTTAAAACCACCCTCCAG
zHDAC6_H574A_F	CGTCCTCCGGGTCATGCTGCTGAGAAAGACAC
zHDAC6_H574_R	GTGTCTTTCTCAGCAGCATGACCCGGAGGACG

Table S3. Data collection and refinement statistics.

**Data collection statistics**	
PDB code	8BJK
Wavelength (Å)	1.34
Space group	P 2 21 21
Unit-cell parameters *a, b, c* (Å)	51.49, 83.78, 94.33
Resolution limits (Å)	62.64-1.35 (1.37-1.35)
Number of unique reflections	90048 (4356)
Redundancy	15 (8.9)
Completeness (%)	99.9 (98.7)
$I/\sigma I$	17.9 (0.7)
R_{pim}	0.032 (0.923)
CC1/2	0.99 (0.30)
**Refinement**	
Resolution limits (Å)	62.64 – 1.35 (1.39 – 1.35)
Total number of reflections	85454 (6545)
Number of reflections in working set	80923 (6218)
Number of reflections in test set	4531 (327)
R/R_{free} (%)	17.0/20.7 (33.3/34.0)
Total number of non-H atoms	3231
Number of non-H protein atoms	2831
Number inhibitor molecules	1
Number of water molecules	374
Average B-factor (Å^2)	15.1
Protein atoms	13.5

Waters	27.5
Inhibitor molecule	10.1
**[&]Ramachandran Plot (%)**	
Most favored	98
Additionally allowed	2
Disallowed	0
**R.m.s. deviations:**	
bond lengths (Å)	0.013
bond angles (°)	1.45
planarity (Å)	0.009
chiral centers ($Å^3$)	0.096
Missing residues	AA 440-441,770-773

* Values in parenthesis are for the highest resolution shells.

[&] Structures were analyzed using the MolProbity 4.02b-467.

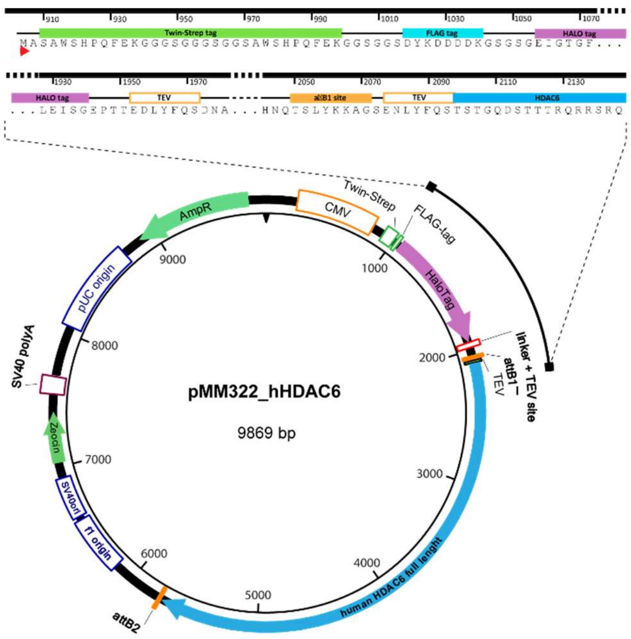

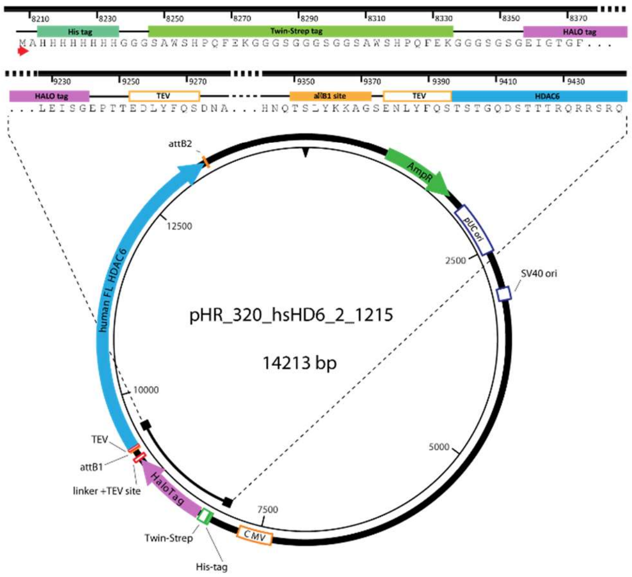

Figure S1: Expression plasmids. **Panel A**: An example of plasmid used for expression of HDACs in mammalian HEK293T cells. HDAC sequences are N-terminally fused to the TEV-cleavable TwinStrep-FLAG-HALO tag. **Panel B**: Schematic representation of a lentiviral expression vector used to generate HEK293 cells stably expressing human HDAC6. The HDAC6 sequence is N-terminally flanked by the TEV-cleavable His-TwinStrep-HALO tag.

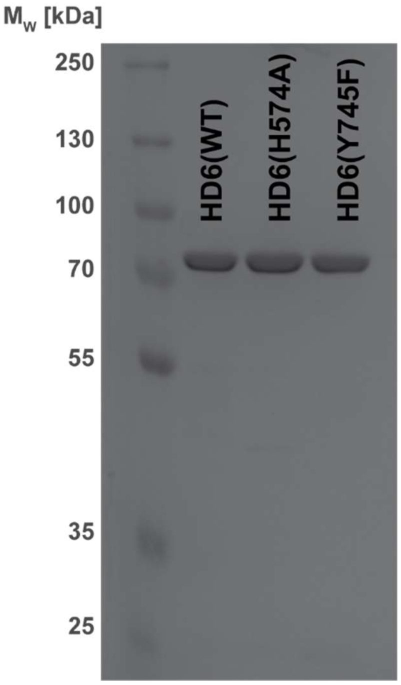

Figure S2: SDS-PAGE analysis of purified His-MBP-zHDAC6 variants. Purified proteins (1 µg) were resolved by SDS-PAGE in 10% polyacrylamide gel that was then stained by Coomassie blue.

N-(3-Chlorophenyl)methanesulfonamide (1)

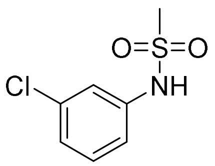

3-Chloroaniline (1.65 mL, 15.6 mmol, 1.0 equiv.) and pyridine (1.40 mL, 17.3 mmol, 1.1 equiv.) were dissolved in anhydrous DCM (40 mL). Methanesulfonyl chloride (1.46 mL, 18.8 mmol, 1.2 equiv.) was added, and the resulting mixture was stirred at room temperature for 2 h under an inert atmosphere. The reaction mixture was extracted with 1M aq. HCl (40 mL), and the organic phase was dried over Na_2SO_4. Volatiles were evaporated, and the residue was recrystallized from a toluene/cyclohexane mixture (2:1) to afford 2.80 g (87%) of *N*-(3-chlorophenyl)methanesulfonamide (**1**) as pink crystals. The ^{1}H NMR spectrum was in agreement with the previously published data.[1]

^{1}H NMR (401 MHz, $CDCl_3$): δ 7.29 (t, *J* = 8.1 Hz, 1H), 7.26 (t, *J* = 2.1 Hz, 1H), 7.19-7.15 (m, 1H), 7.13-7.09 (m, 1H), 6.67 (br s, 1H), 3.05 (s, 3H).

Methyl 6-(bromomethyl)nicotinate (2)

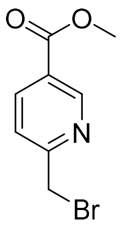

Methyl 6-methylnicotinate (1.40 g, 9.26 mmol, 1.0 equiv.) and *N*-bromosuccinimide (1.81 g, 10.2 mmol, 1.1 equiv.) were dissolved in anhydrous benzene (30 mL). Dibenzoyl peroxide (0.11 g, 0.46 mmol, 0.05 equiv.) was added, and the resulting mixture was heated to 80 °C for 18 h under an inert atmosphere. Volatiles were evaporated, and the residue was subjected to flash column chromatography (silica gel 60, 70-230 mesh, solvent: cyclohexane/ethyl acetate 4:1) to afford 310 mg (15%) of methyl 6-(bromomethyl)nicotinate (**2**) as a white solid. The ^{1}H NMR spectrum was in agreement with the previously published data.[1]

^{1}H NMR (401 MHz, $CDCl_3$): δ 9.17 (dd, *J* = 0.9, 2.2 Hz, 1H), 8.30 (dd, *J* = 2.2, 8.1 Hz, 1H), 7.54 (dd, *J* = 0.9, 8.1 Hz, 1H), 4.58 (s, 2H), 3.96 (s, 3H).

Methyl 6-((*N*-(3-chlorophenyl)methylsulfonamido)methyl)nicotinate (3)

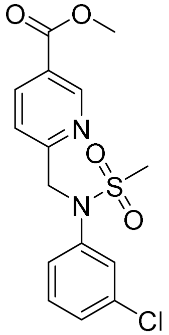

N-(3-Chlorophenyl)methanesulfonamide (**1**, 0.20 g, 1.00 mmol, 1.0 equiv.) was dissolved in anhydrous DMF (8 mL), and 60% NaH in mineral oil (48 mg, 1.20 mmol, 1.2 equiv.) was added at 0 °C. After 30 min, compound **2** (0.23 g, 1.00 mmol, 1.0 equiv.) was added, and the reaction mixture was allowed to warm to room temperature and stirred overnight. After 16 hours, volatiles were evaporated, and the residue was subjected to flash column chromatography (silica gel 60, 70-230 mesh, solvent: dichloromethane/methanol 50:1) to afford 330 mg (93%) of methyl 6-((*N*-(3-chlorophenyl)methylsulfonamido)methyl)nicotinate (**3**) as a light yellow solid.

^{1}H NMR (401 MHz, CDCl$_3$): δ 9.14 – 9.10 (m, 1H), 8.31 – 8.22 (m, 1H), 7.50 (d, *J* = 8.20 Hz, 1H), 7.45 – 7.40 (m, 1H), 7.32 – 7.21 (m, 3H), 5.07 (s, 2H), 3.94 (s, 3H), 3.06 (s, 3H).

^{13}C NMR (101 MHz, CDCl$_3$): δ 165.6, 160.7, 150.6, 140.8, 138.2, 135.1, 130.5, 128.4, 128.1, 126.1, 125.3, 122.0, 56.4, 52.6, 38.8.

ESI MS: 355.1 ([M + H]$^+$).

HR ESI MS: calcd for $C_{15}H_{16}O_4N_2ClS$ 355.05138; found 355.05133.

***N*-(3-Chlorophenyl)-*N*-((5-(hydrazinecarbonyl)pyridin-2-yl)methyl)methanesulfonamide (4)**

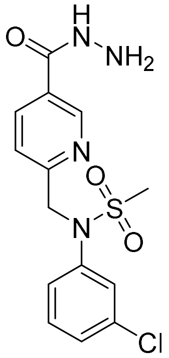

Compound **3** (0.35 g, 1.00 mmol, 1.0 equiv.) was dissolved in ethanol (10 mL), and hydrazine hydrate (50% aq. solution, 0.53 mL, 10 equiv.) was added. The resulting mixture was stirred at 80 °C for 16 h. Volatiles were then removed under reduced pressure, and the product was purified by preparative HPLC with a gradient of 0-50% acetonitrile in H_2O (with 0.1% TFA). The fractions containing the desired

product were collected and lyophilized to afford 320 mg (91%) of *N*-(3-chlorophenyl)-*N*-((5-(hydrazinecarbonyl)pyridin-2-yl)methyl)methanesulfonamide (**4**) as a white solid.

^{1}H NMR (401 MHz, CD_3OD): δ 8.92 (dd, *J* = 0.8, 2.2 Hz, 1H), 8.22 (dd, *J* = 2.3, 8.2 Hz, 1H), 7.67 (d, *J* = 8.2 Hz, 1H), 7.51 (t, *J* = 1.9 Hz, 1H), 7.43 – 7.38 (m, 1H), 7.33 (t, *J* = 7.9 Hz, 1H), 7.31 – 7.26 (m, 1H), 5.11 (s, 2H), 3.11 (s, 3H).

^{13}C NMR (101 MHz, CD_3OD): δ 166.6, 162.3, 149.3, 142.4, 137.8, 135.7, 131.6, 129.6, 129.1, 127.6, 127.4, 123.9, 56.8, 38.1.

ESI MS: 355.1 ($[M + H]^+$).

HR ESI MS: calcd for $C_{14}H_{16}O_3N_4ClS$ 355.06262; found 355.06241.

_N_-(3-Chlorophenyl)-_N_-((5-(2-(2,2,2-trifluoroacetyl)hydrazine-1-carbonyl)pyridin-2-yl)methyl)methanesulfonamide (5)

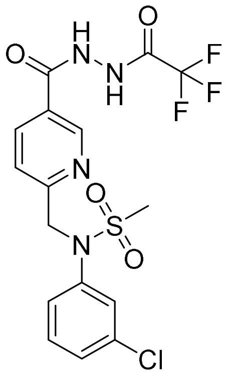

Compound **4** (36 mg, 0.10 mmol, 1.0 equiv.) and triethylamine (42 µL, 0.30 mmol, 3 equiv.) were dissolved in anhydrous DCM (2 mL) at 0 °C, and trifluoroacetic anhydride (16 µL, 0.11 mmol, 1.1 equiv.) was added. The resulting mixture was stirred at 0 °C for 1 h. Volatiles were then removed under reduced pressure, and the product was purified by preparative HPLC with a gradient of 15-50% acetonitrile in H_2O (with 0.1% TFA). The fractions containing the desired product were collected and lyophilized to afford 35 mg (78%) of *N*-(3-chlorophenyl)-*N*-((5-(2-(2,2,2-trifluoroacetyl)hydrazine-1-carbonyl)pyridin-2-yl)methyl)methanesulfonamide (**5**) as a white solid.

^{1}H NMR (401 MHz, CD_3OD): δ 8.93 (d, *J* = 1.7 Hz, 1H), 8.24 (dd, *J* = 2.3, 8.2 Hz, 1H), 7.68 (d, *J* = 8.2 Hz, 1H), 7.53 (t, *J* = 1.8 Hz, 1H), 7.40 (dt, *J* = 1.8, 7.8 Hz, 1H), 7.34 (t, *J* = 7.8 Hz, 1H), 7.30 (dt, *J* = 1.7, 7.9 Hz, 1H), 5.12 (s, 2H), 3.12 (s, 3H).

^{13}C NMR (101 MHz, CD_3OD): δ 166.3, 161.7, 158.2 (q, $J_{C,F}$ = 37.6 Hz), 149.0, 142.3, 138.1, 135.7, 131.6, 129.6, 129.2, 128.5, 127.6, 124.0, 117.4 (q, $J_{C,F}$ = 285.7 Hz), 56.7, 38.2.

ESI MS: 451.0 ($[M + H]^+$).

HR ESI MS: calcd for $C_{16}H_{15}O_4N_4ClF_3S$ 451.04491; found 451.04459.

***N*-(3-Chlorophenyl)-*N*-((5-(2-(2,2-difluoroacetyl)hydrazine-1-carbonyl)pyridin-2-yl)methyl)methanesulfonamide (6)**

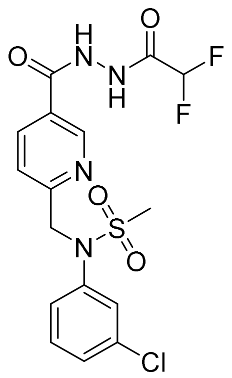

Compound **4** (36 mg, 0.10 mmol, 1.0 equiv.) and triethylamine (42 µL, 0.30 mmol, 3 equiv.) were dissolved in anhydrous DCM (2 mL) at 0 °C, and difluoroacetic anhydride (12.4 µL, 0.10 mmol, 1.0 equiv.) was added. The resulting mixture was stirred at 0 °C for 1 h. Volatiles were then removed under reduced pressure, and the product was purified by preparative HPLC with a gradient of 15-50% acetonitrile in H_2O (with 0.1% TFA). The fractions containing the desired product were collected and lyophilized to afford 37 mg (85%) of *N*-(3-chlorophenyl)-*N*-((5-(2-(2,2-difluoroacetyl)hydrazine-1-carbonyl)pyridin-2-yl)methyl)methanesulfonamide (**6**) as a white solid.

^{1}H NMR (401 MHz, CD_3OD): δ 8.93 (d, *J* = 1.6 Hz, 1H), 8.25 (dd, *J* = 2.3, 8.2 Hz, 1H), 7.68 (d, *J* = 8.2 Hz, 1H), 7.52 (t, *J* = 1.8 Hz, 1H), 7.43 – 7.38 (m, 1H), 7.32 – 7.27 (m, 1H), 7.30 (dt, *J* = 1.7, 7.9 Hz, 1H), 6.23 (t, *J* = 53.2 Hz, 1H), 5.12 (s, 2H), 3.11 (s, 3H).

^{13}C NMR (101 MHz, CD_3OD): δ 166.5, 164.1 (t, $J_{C,F}$ = 25.6 Hz), 161.5, 149.0, 142.3, 138.1, 135.7, 131.6, 129.6, 129.2, 128.7, 127.6, 124.0, 109.8 (t, $J_{C,F}$ = 249.0 Hz), 56.7, 38.2.

ESI MS: 433.1 ($[M + H]^+$).

HR ESI MS: calcd for $C_{16}H_{16}O_4N_4ClF_2S$ 433.05434; found 433.05401.

***N*-(3-Chlorophenyl)-*N*-((5-(5-(trifluoromethyl)-1,3,4-oxadiazol-2-yl)pyridin-2-yl)methyl)methanesulfonamide (7)**

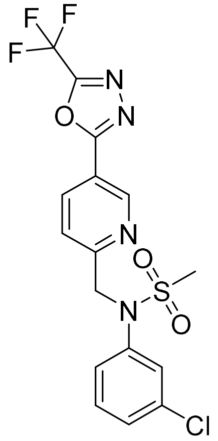

Compound **5** (28 mg, 0.06 mmol, 1.0 equiv.) and methyl *N*-(triethylammoniumsulfonyl)carbamate (Burgess reagent, 44 mg, 0.19 mmol, 3 equiv.) were dissolved in anhydrous THF (0.5 mL) at room temperature. The resulting mixture was stirred at 50 °C for 16 h. Volatiles were then removed under reduced pressure, and the product was purified by preparative HPLC with a gradient of 20-60% acetonitrile in H_2O (with 0.1% TFA). The fractions containing the desired product were collected and lyophilized to afford 18 mg (67%) of *N*-(3-chlorophenyl)-*N*-((5-(5-(trifluoromethyl)-1,3,4-oxadiazol-2-yl)pyridin-2-yl)methyl)methanesulfonamide (**7**) as a white solid.

^{1}H NMR (401 MHz, $CDCl_3$): δ 9.24 (d, J = 1.6 Hz, 1H), 8.48 (dd, J = 2.2, 8.3 Hz, 1H), 7.80 (d, J = 8.3 Hz, 1H), 7.46 – 7.42 (m, 1H), 7.35 – 7.27 (m, 3H), 5.17 (s, 2H), 3.07 (s, 3H).

^{13}C NMR (101 MHz, $CDCl_3$): δ 164.0, 161.0, 155.5 (q, $J_{C,F}$ = 45.0 Hz), 147.1, 140.4, 136.8, 135.4, 130.8, 128.8, 128.1, 125.9, 123.5, 118.7, 116.2 (q, $J_{C,F}$ = 273.0 Hz), 55.6, 38.3.

ESI MS: 433.0 ([M + H]$^+$).

HR ESI MS: calcd for $C_{16}H_{13}O_3N_4ClF_3S$ 433.03435; found 433.03396.

***N*-(3-Chlorophenyl)-*N*-((5-(5-(difluoromethyl)-1,3,4-oxadiazol-2-yl)pyridin-2-yl)methyl)methanesulfonamide (8)**

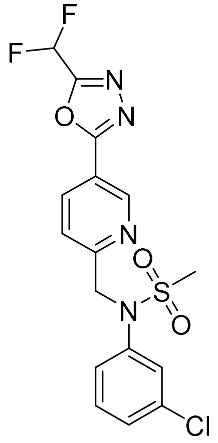

Compound **6** (24 mg, 0.05 mmol, 1.0 equiv.) and methyl *N*-(triethylammoniumsulfonyl)carbamate (Burgess reagent, 39 mg, 0.16 mmol, 3 equiv.) were dissolved in anhydrous THF (0.5 mL) at room temperature. The resulting mixture was stirred at 50 °C for 16 h. Volatiles were then removed under reduced pressure, and the product was purified by preparative HPLC with a gradient of 20-60% acetonitrile in H_2O (with 0.1% TFA). The fractions containing the desired product were collected and lyophilized to afford 18 mg (78%) of *N*-(3-chlorophenyl)-*N*-((5-(5-(difluoromethyl)-1,3,4-oxadiazol-2-yl)pyridin-2-yl)methyl)methanesulfonamide (**8**) as a white solid.

^{1}H NMR (401 MHz, $CDCl_3$): δ 9.24 (d, J = 1.6 Hz, 1H), 8.37 (dd, J = 2.2, 8.2 Hz, 1H), 7.64 (d, J = 8.2 Hz, 1H), 7.47 – 7.42 (m, 1H), 7.35 – 7.23 (m, 3H), 6.93 (t, J = 51.6 Hz, 1H), 5.11 (s, 2H), 3.07 (s, 3H).

^{13}C NMR (101 MHz, $CDCl_3$): δ 164.1, 160.9, 158.7 (t, $J_{C,F}$ = 29.0 Hz), 148.0, 140.7, 135.7, 135.2, 130.6, 128.5, 128.1, 126.0, 122.7, 118.5, 105.8 (t, $J_{C,F}$ = 241.0 Hz), 56.4, 38.7.

ESI MS: 415.0 ($[M + H]^+$).

HR ESI MS: calcd for $C_{19}H_{26}O_5N_2Na$ 415.04377; found 415.04372.

***N*-(3-Chlorophenyl)-*N*-((5-(5-(fluoromethyl)-1,3,4-oxadiazol-2-yl)pyridin-2-yl)methyl)methanesulfonamide (9)**

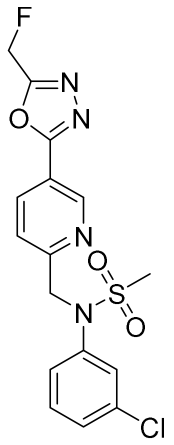

Compound **4** (36 mg, 0.10 mmol, 1.0 equiv.) and triethylamine (42 µL, 0.30 mmol, 3 equiv.) were dissolved in anhydrous DCM (2 mL) at 0 °C, and fluoroacetic anhydride (27.6 mg, 0.20 mmol, 2.0 equiv.) was added. The resulting mixture was stirred at room temperature for 16 h. Volatiles were then removed under reduced pressure, the residue was dissolved in anhydrous THF (0.5 mL), and methyl *N*-(triethylammoniumsulfonyl)carbamate (Burgess reagent, 71 mg, 0.3 mmol, 3 equiv.) was added. The resulting mixture was stirred at 50 °C for 16 h. Volatiles were then removed under reduced pressure, and the product was purified by preparative HPLC with a gradient of 20-60% acetonitrile in H_2O (with 0.1% TFA). The fractions containing the desired product were collected and lyophilized to afford 25 mg (63%) of *N*-(3-chlorophenyl)-*N*-((5-(5-(fluoromethyl)-1,3,4-oxadiazol-2-yl)pyridin-2-yl)methyl)methanesulfonamide (**9**) as a white solid.

^{1}H NMR (401 MHz, $CDCl_3$): δ 10.62 (br s, 1H), 9.23 (d, *J* = 2.6, 1H), 8.58 (dd, *J* = 2.1, 8.3 Hz, 1H), 7.91 (d, *J* = 8.3 Hz, 1H), 7.44 (q, *J* = 1.5 Hz, 1H), 7.34 – 7.28 (m, 3H), 5.63 (d, *J* = 46.8 Hz, 2H), 5.23 (s, 2H), 3.06 (s, 3H).

^{13}C NMR (101 MHz, $CDCl_3$): δ 163.0, 162.1 (d, $J_{C,F}$ = 20.0 Hz), 159.5, 145.5, 140.2, 137.8, 135.5, 130.9, 129.0, 128.2, 125.9, 124.3, 120.2, 73.1 (d, $J_{C,F}$ = 174.1 Hz), 54.6, 38.0.

ESI MS: 419.0 ($[M + Na]^+$).

HR ESI MS: calcd for $C_{16}H_{15}O_3N_4ClFS$ 397.05319; found 397.05344.

_N_-(3-Chlorophenyl)-_N_-((5-(5-methyl-1,3,4-oxadiazol-2-yl)pyridin-2-yl)methyl)methanesulfonamide (10)

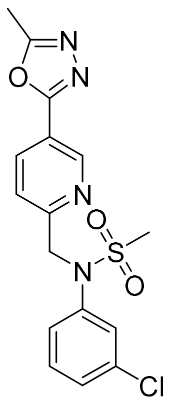

Compound **4** (15 mg, 0.04 mmol, 1.0 equiv.) was dissolved in triethyl orthoacetate (150 µL), and the resulting mixture was stirred at 120 °C for 16 h. Volatiles were then removed under reduced pressure, and the product was purified by preparative HPLC with a gradient of 20-60% acetonitrile in H_2O (with 0.1% TFA). The fractions containing the desired product were collected and lyophilized to afford 12 mg (74%) of *N*-(3-chlorophenyl)-*N*-((5-(5-(methyl)-1,3,4-oxadiazol-2-yl)pyridin-2-yl)methyl)methanesulfonamide (**10**) as a white solid.

^{1}H NMR (401 MHz, $CDCl_3$): δ 9.16 (d, *J* = 1.6 Hz, 1H), 8.48 (dd, *J* = 2.1, 8.3 Hz, 1H), 8.26 (br s, 1H), 7.82 (d, *J* = 8.3 Hz, 1H), 7.46 – 7.42 (m, 1H), 7.35 – 7.27 (m, 3H), 5.19 (s, 2H), 3.06 (s, 3H), 2.67 (s, 3H).

^{13}C NMR (101 MHz, $CDCl_3$): δ 164.9, 162.1, 159.0, 145.6, 140.3, 136.9, 135.4, 130.8, 128.8, 128.2, 125.9, 123.9, 120.5, 55.0, 38.1, 11.2.

ESI MS: 401.0 ($[M + Na]^+$).

HR ESI MS: calcd for $C_{16}H_{16}O_3N_4ClS$ 379.06262; found 379.06276.

References SI

1. Ptacek, J.; Snajdr, I.; Schimer, J.; Kutil, Z.; Mikesova, J.; Baranova, P.; Havlinova, B.; Tueckmantel, W.; Majer, P.; Kozikowski, A.; Barinka, C., Selectivity of Hydroxamate- and Difluoromethyloxadiazole-Based Inhibitors of Histone Deacetylase 6 In Vitro and in Cells. *Int J Mol Sci* **2023,** *24* (5).
