# Supplementary figures and images for: Comprehensive Mechanistic View of the Hydrolysis of Oxadiazole-Based Inhibitors by Histone Deacetylase 6 (HDAC6)
Source: ACS Chem Biol. 2023 Jul 3;18(7):1594–610. doi: 10.1021/acschembio.3c00212 (PMC10367051; doi:10.1021/acschembio.3c00212)

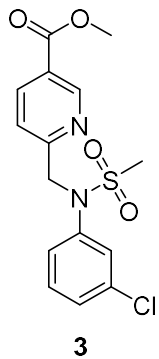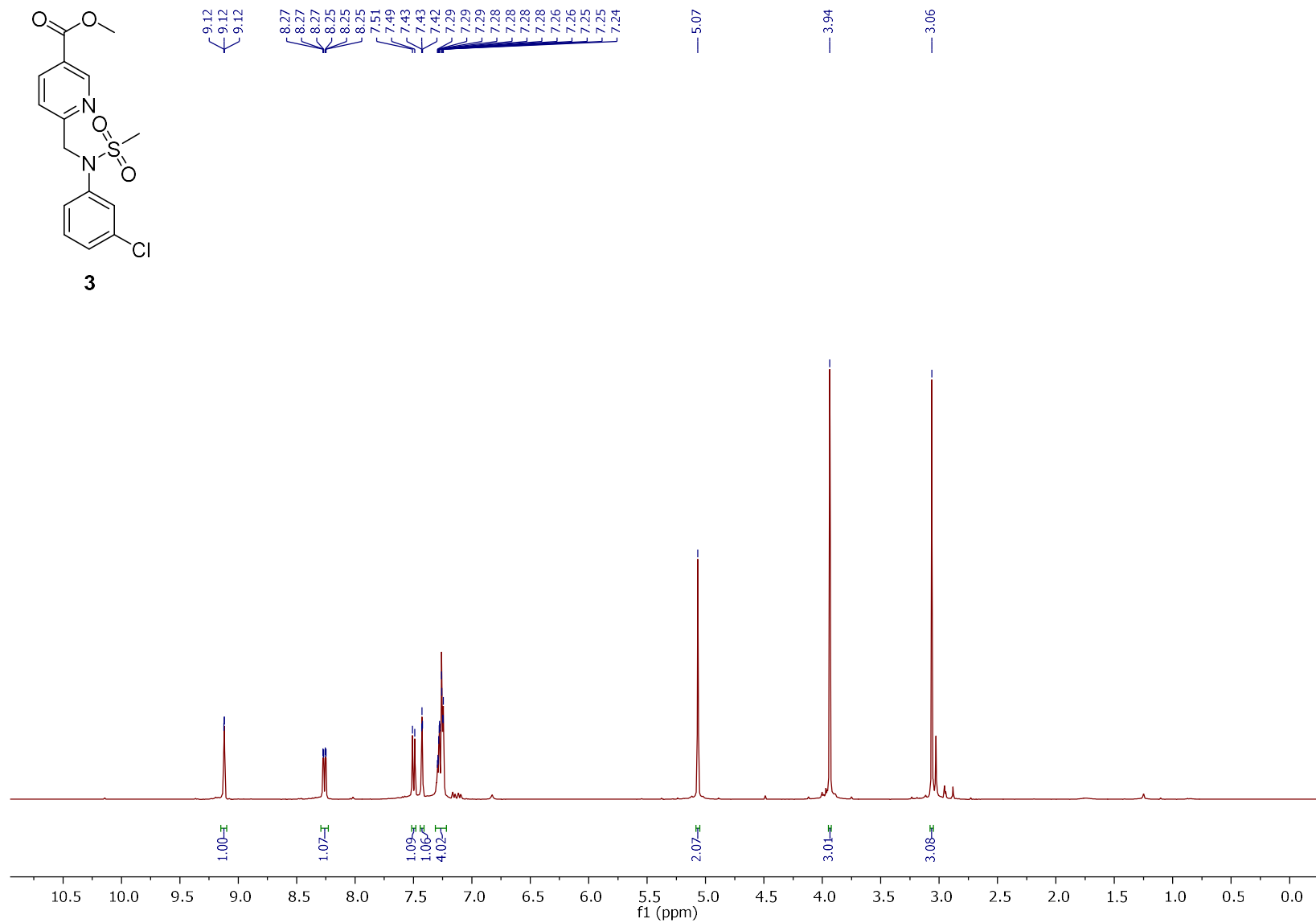

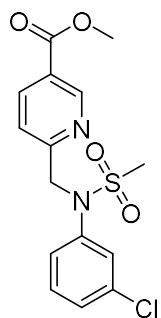

**3**

— 165.56

— 160.70

— 150.64

~ 140.83

~ 138.15

~ 135.10

~ 130.52

~ 128.37

~ 128.11

~ 126.05

~ 125.31

~ 122.03

— 56.42

— 52.59

— 38.76

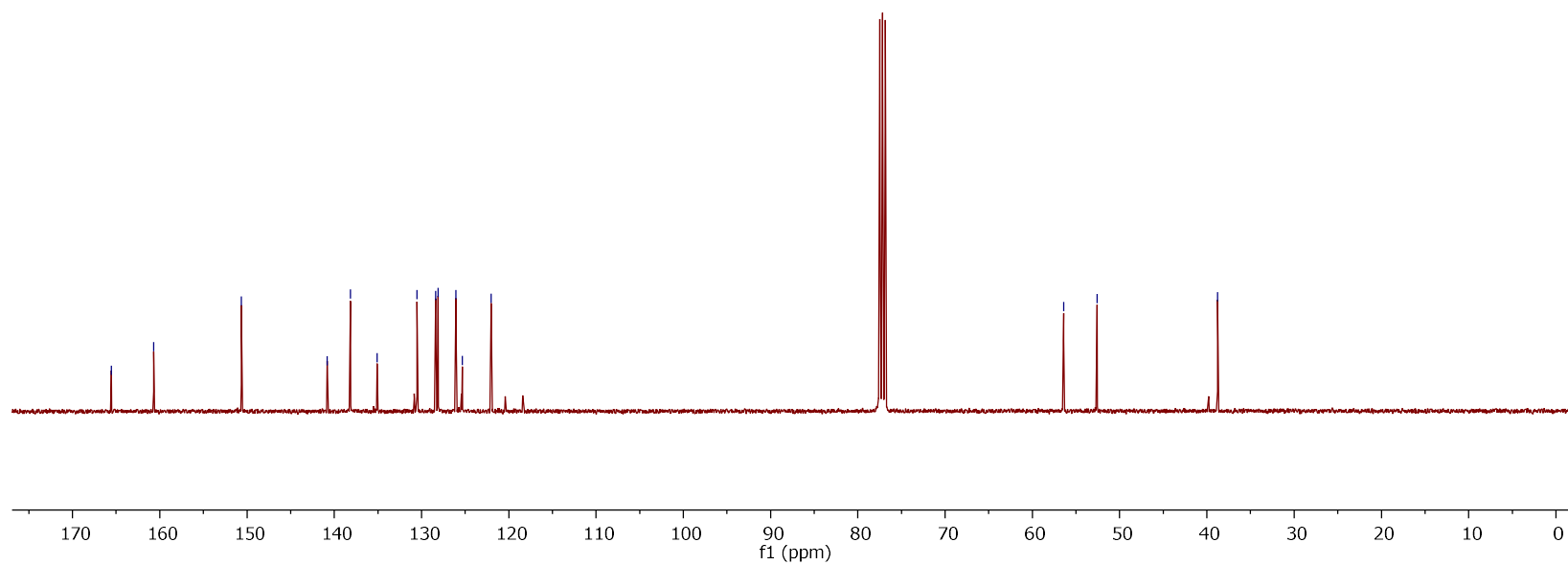

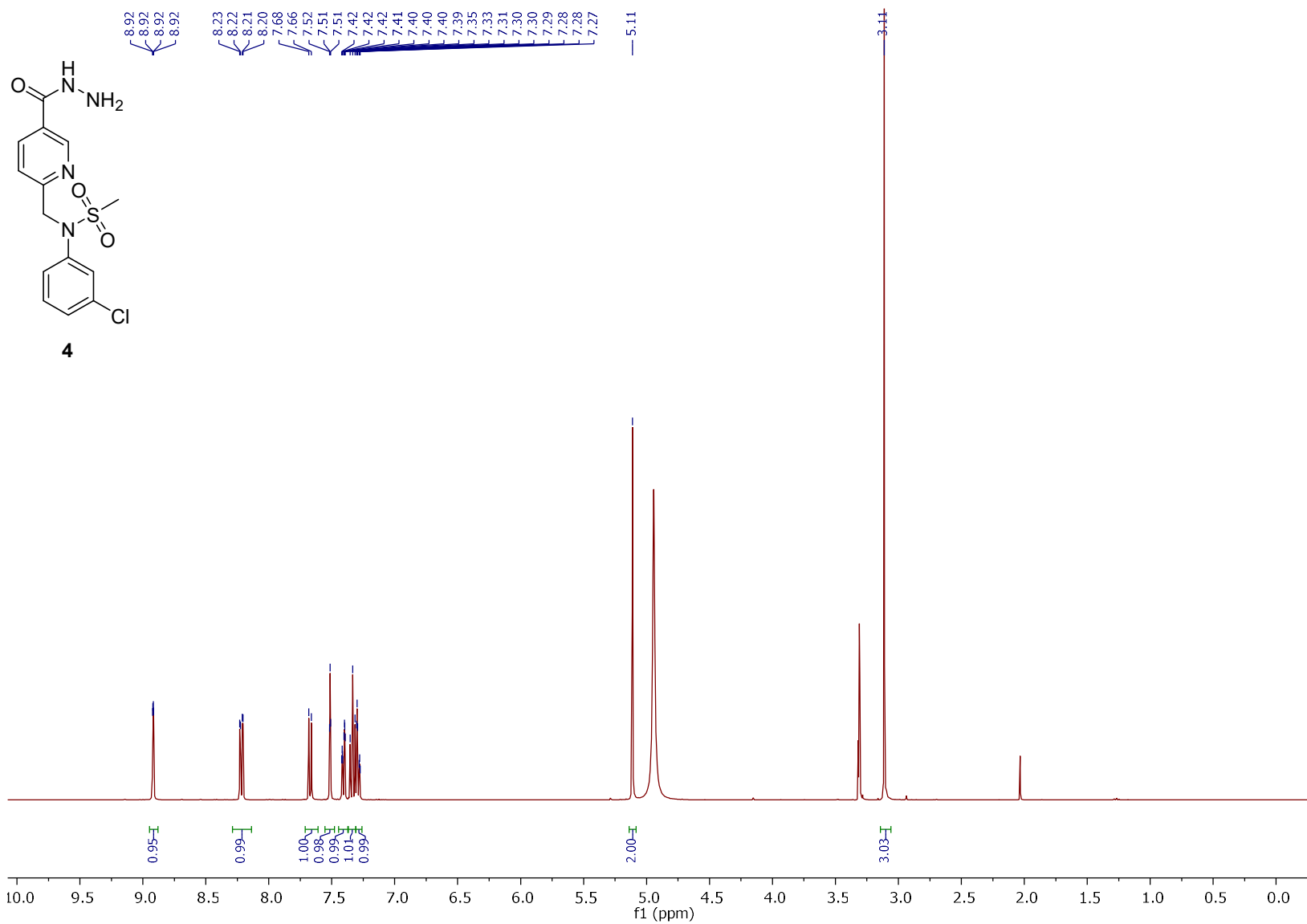

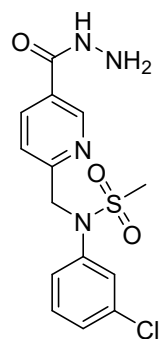

**4**

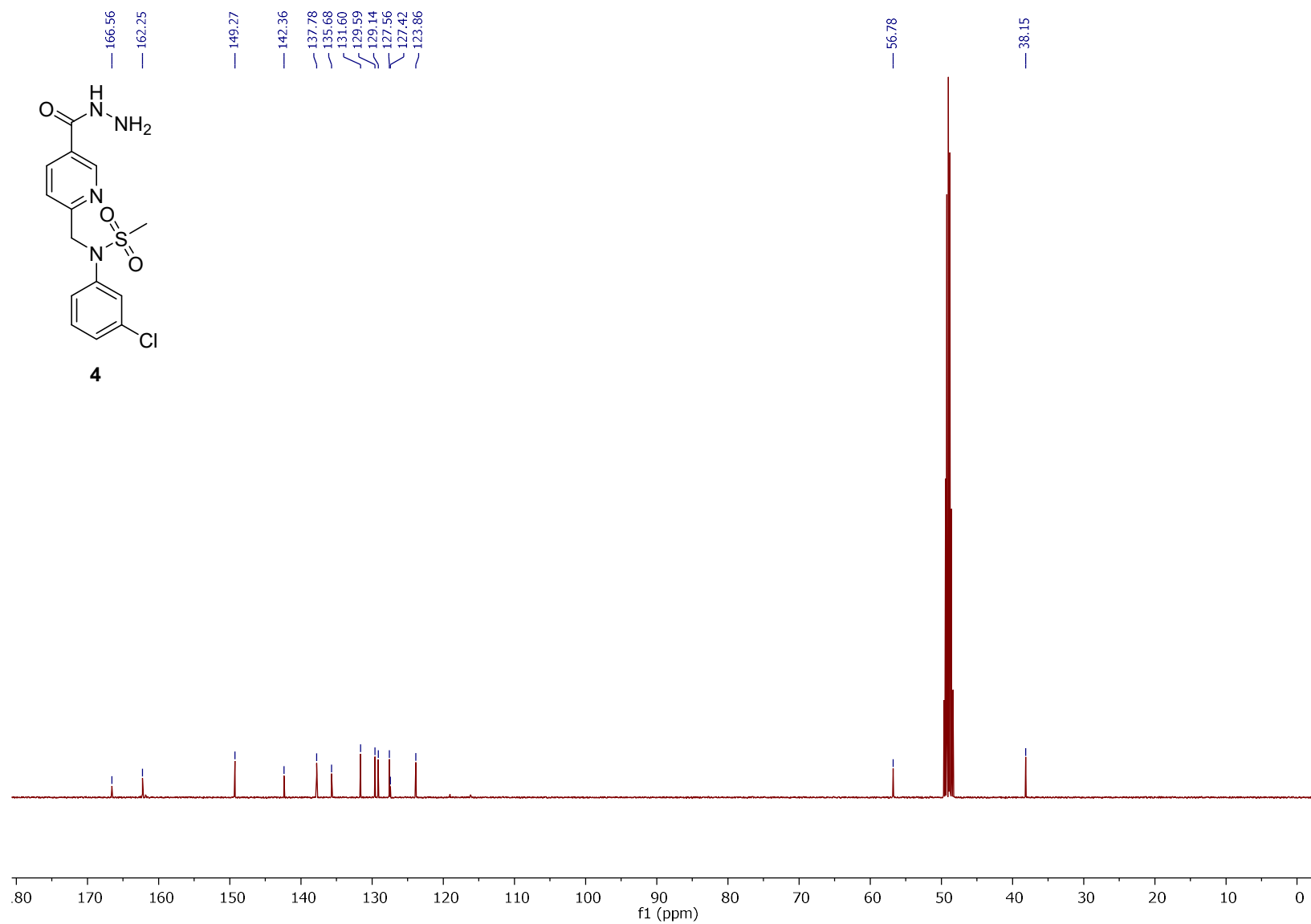

IS-103-012.1.fid

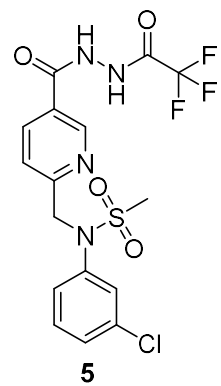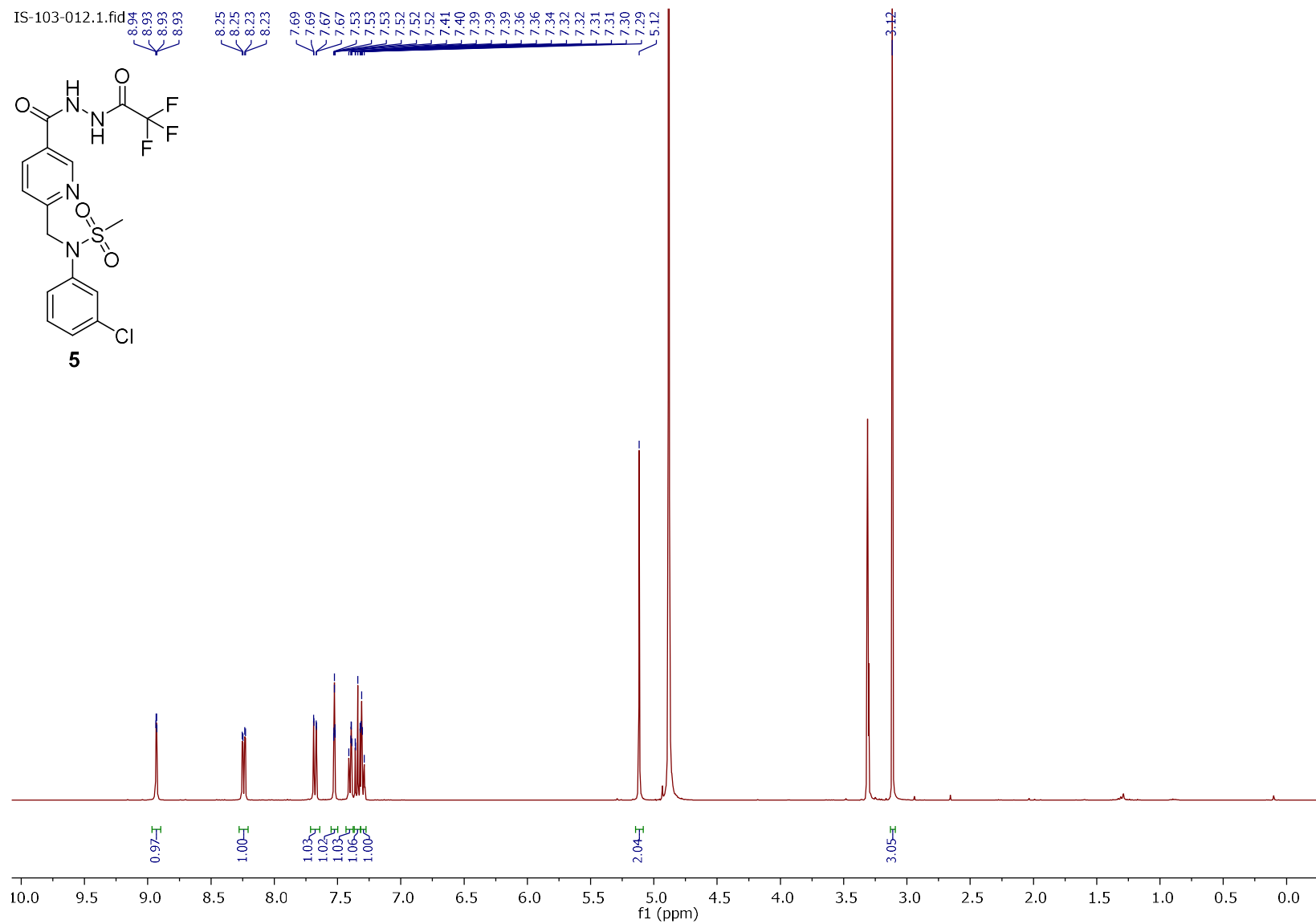

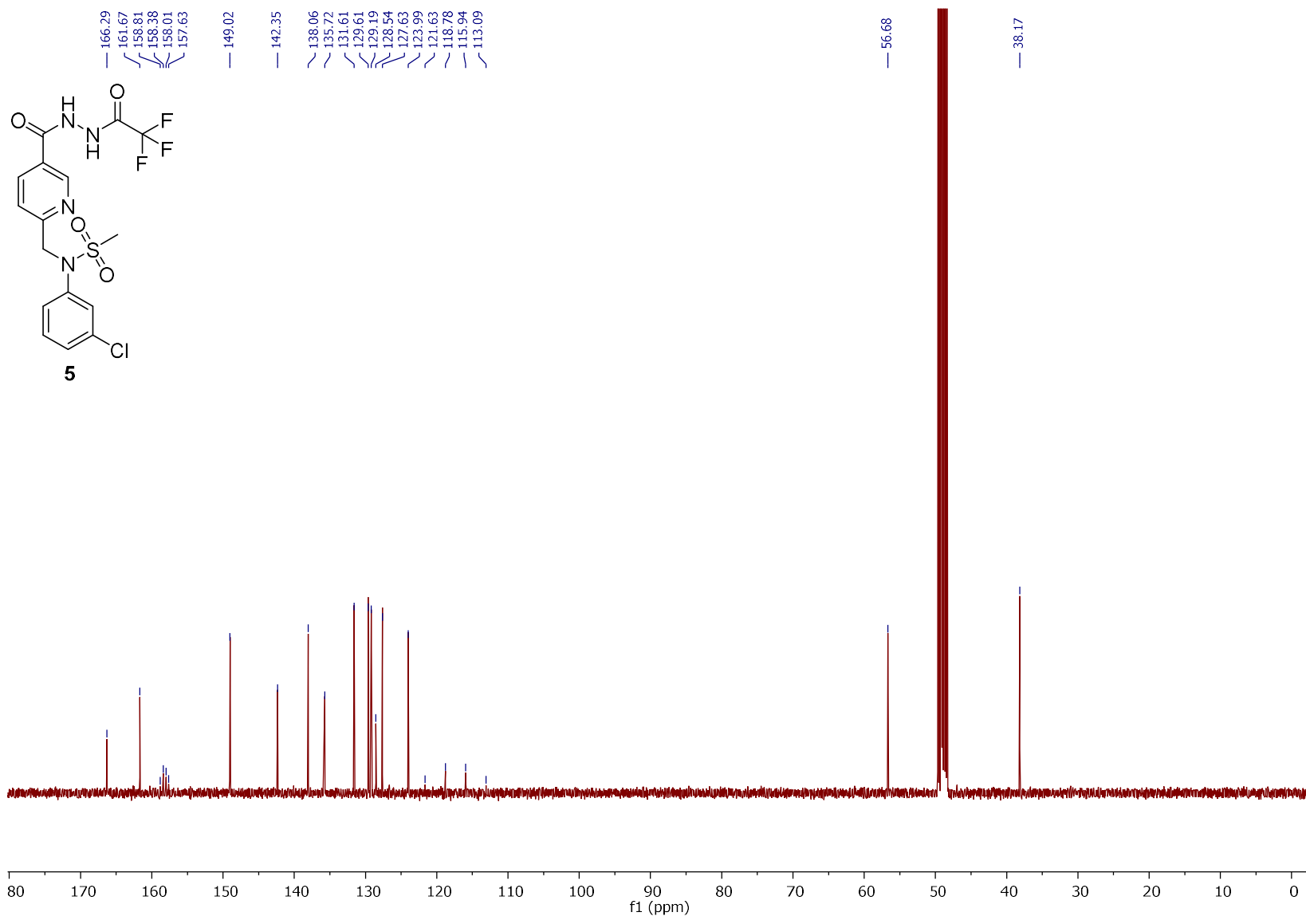

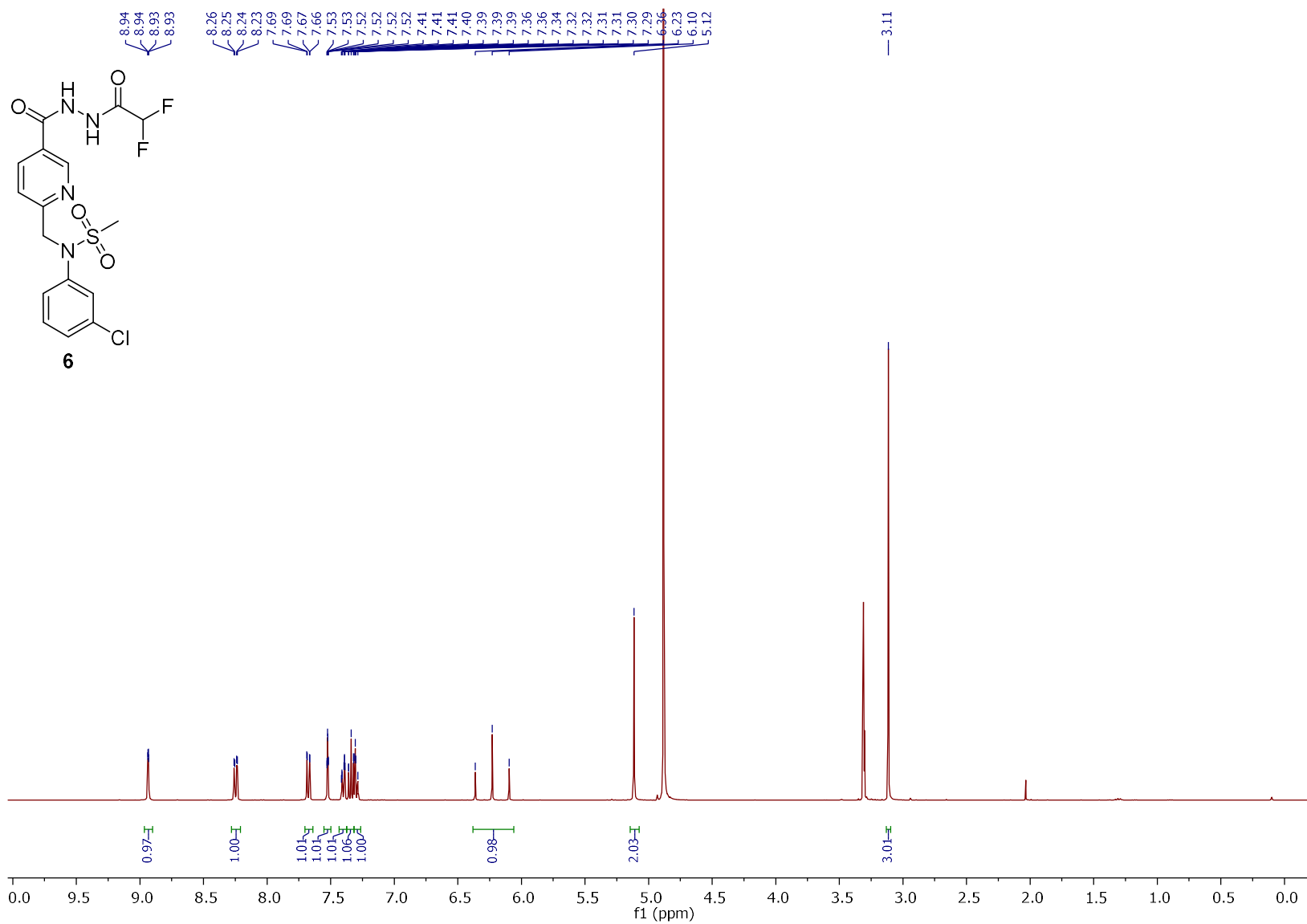

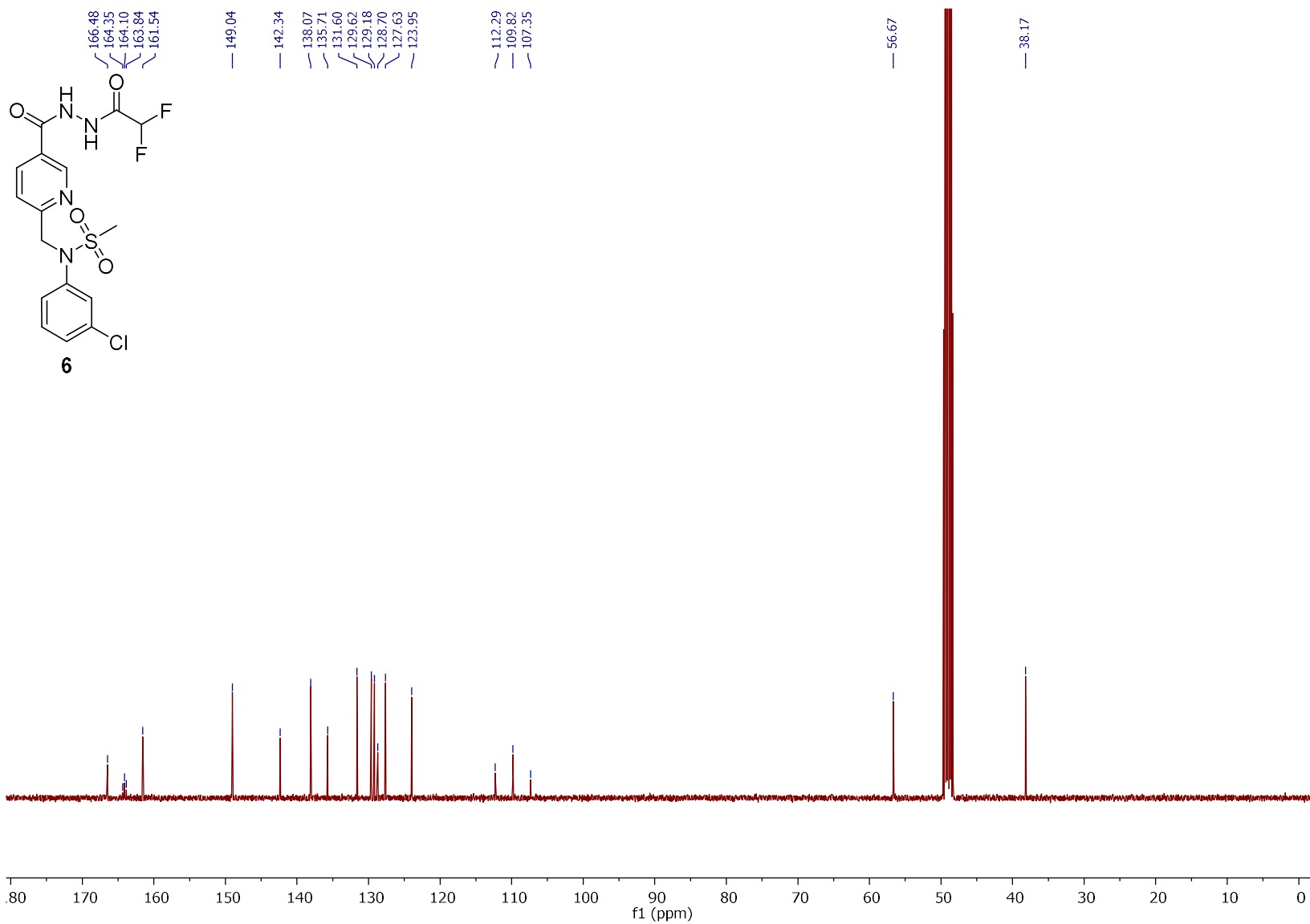

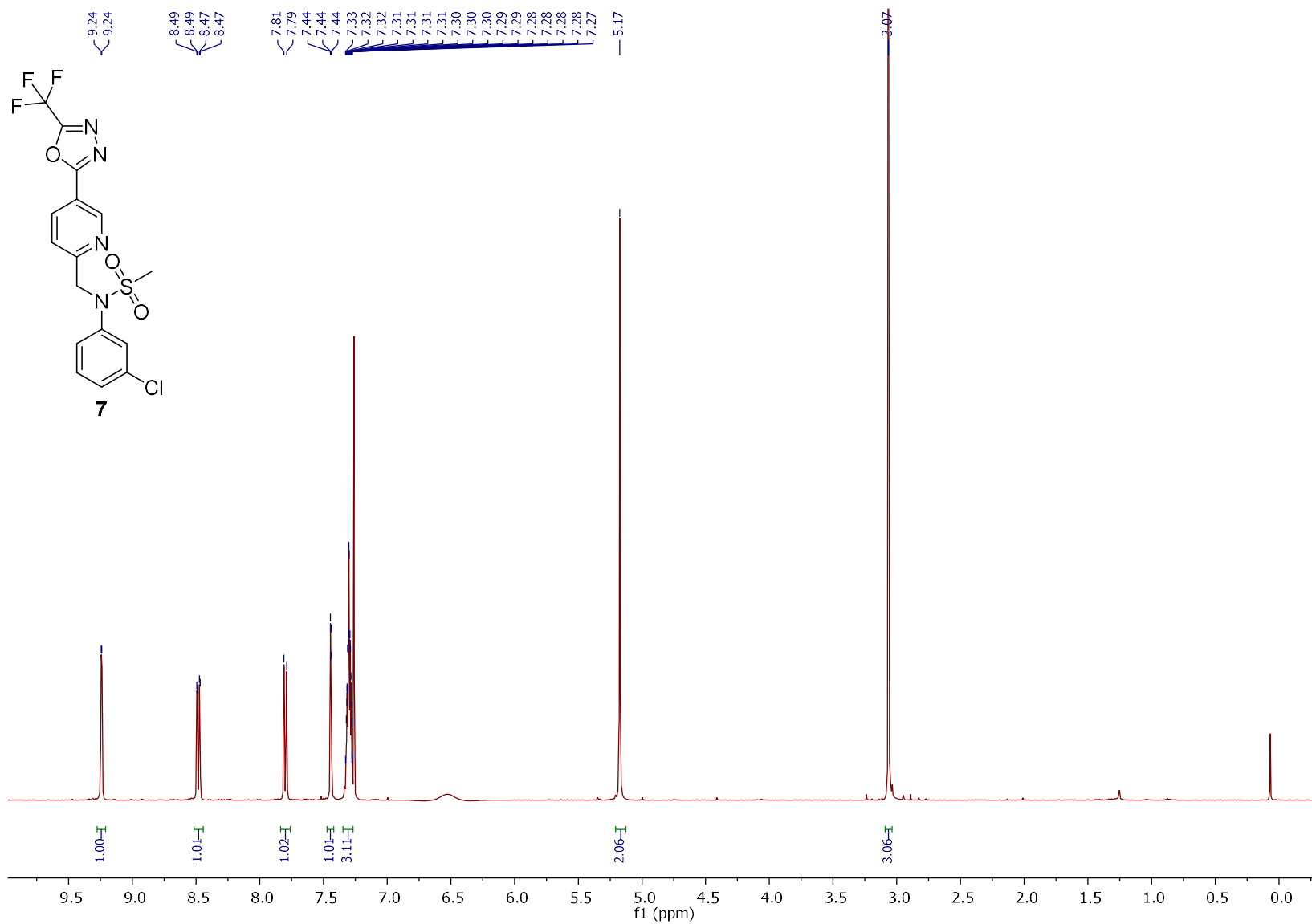

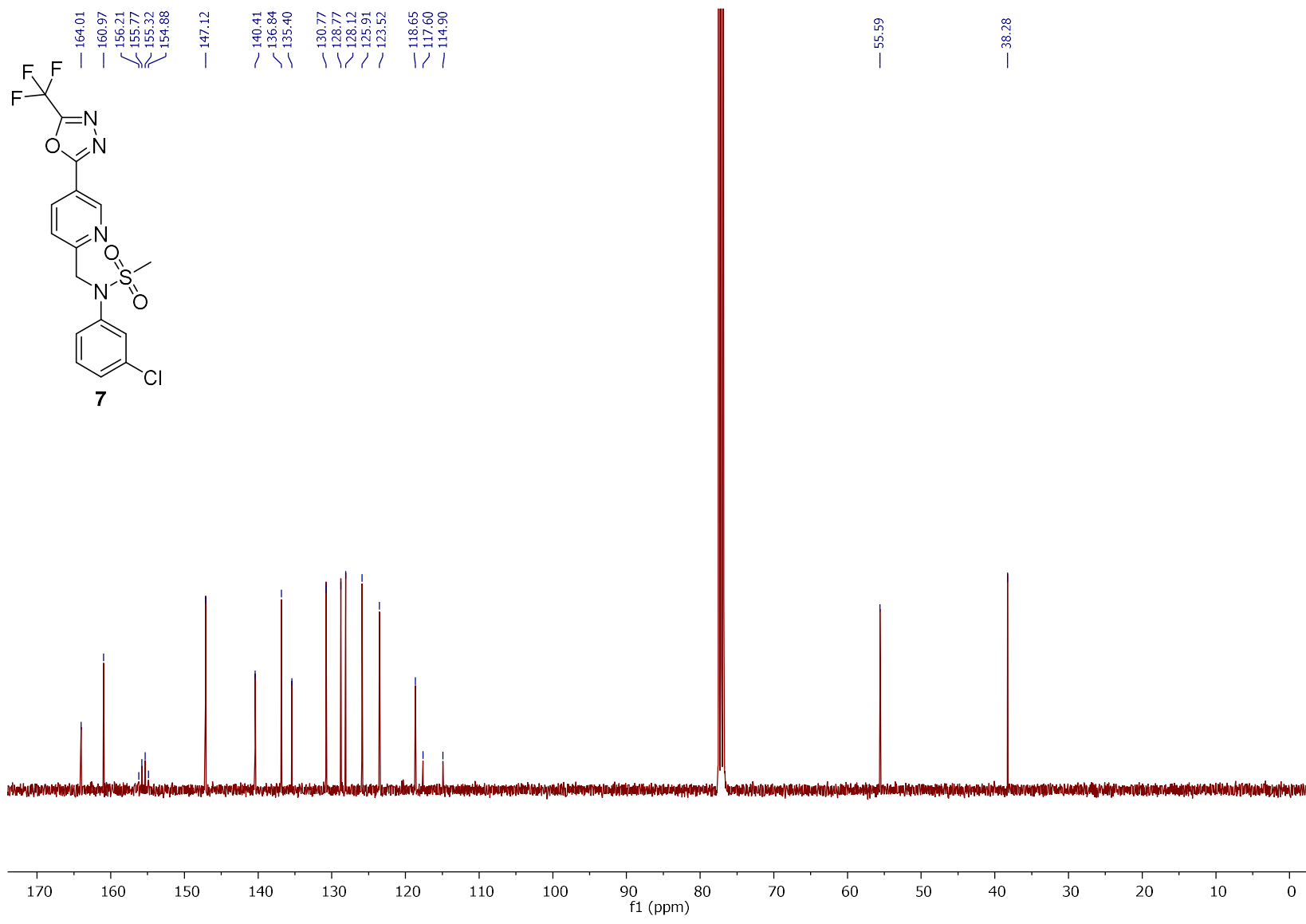

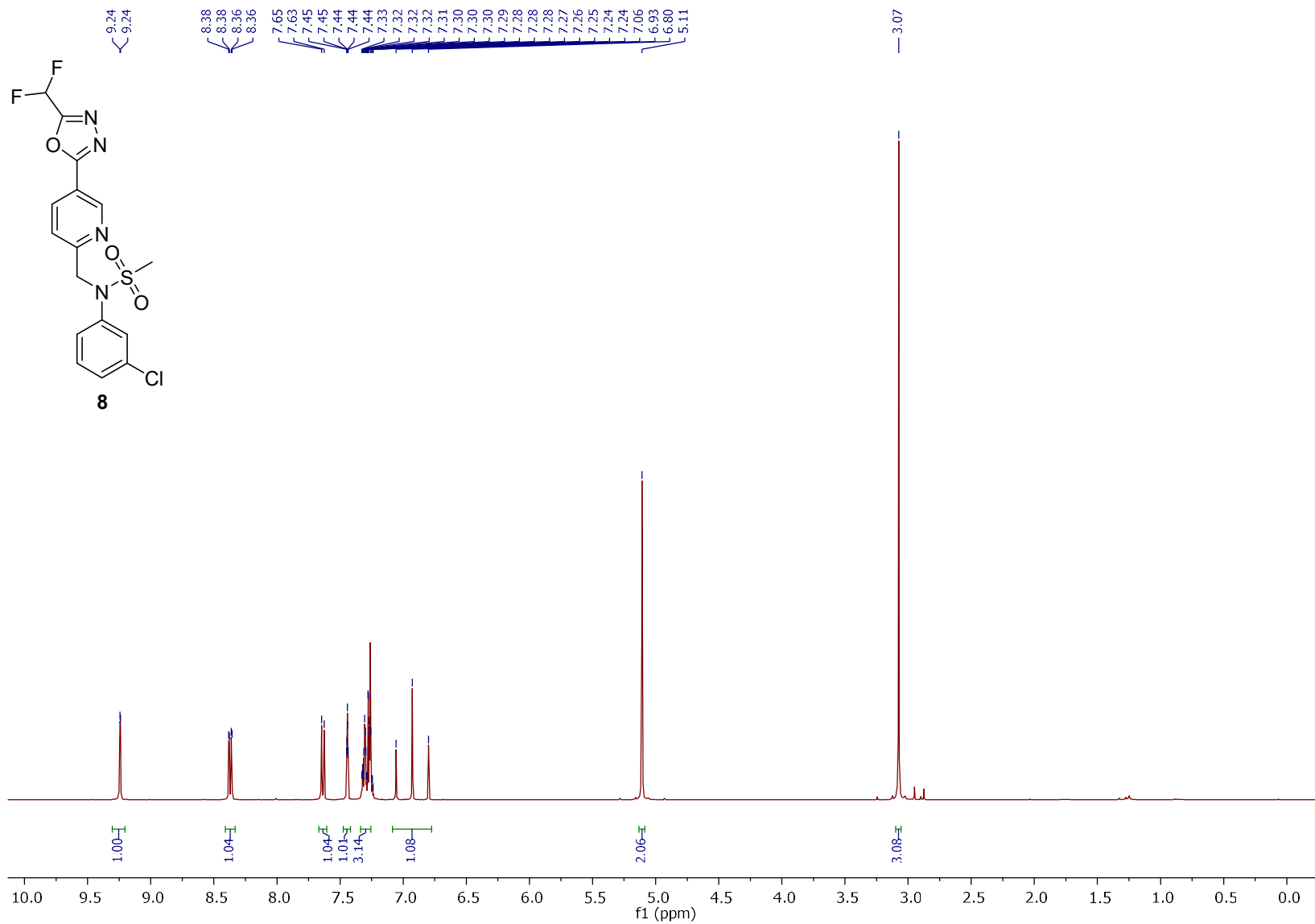

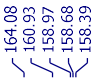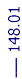

—140.73

135.71

13020

128.47  
128.06

— 122.69

— 118.49

— 108.16

103.37

— 56.40

— 38.68

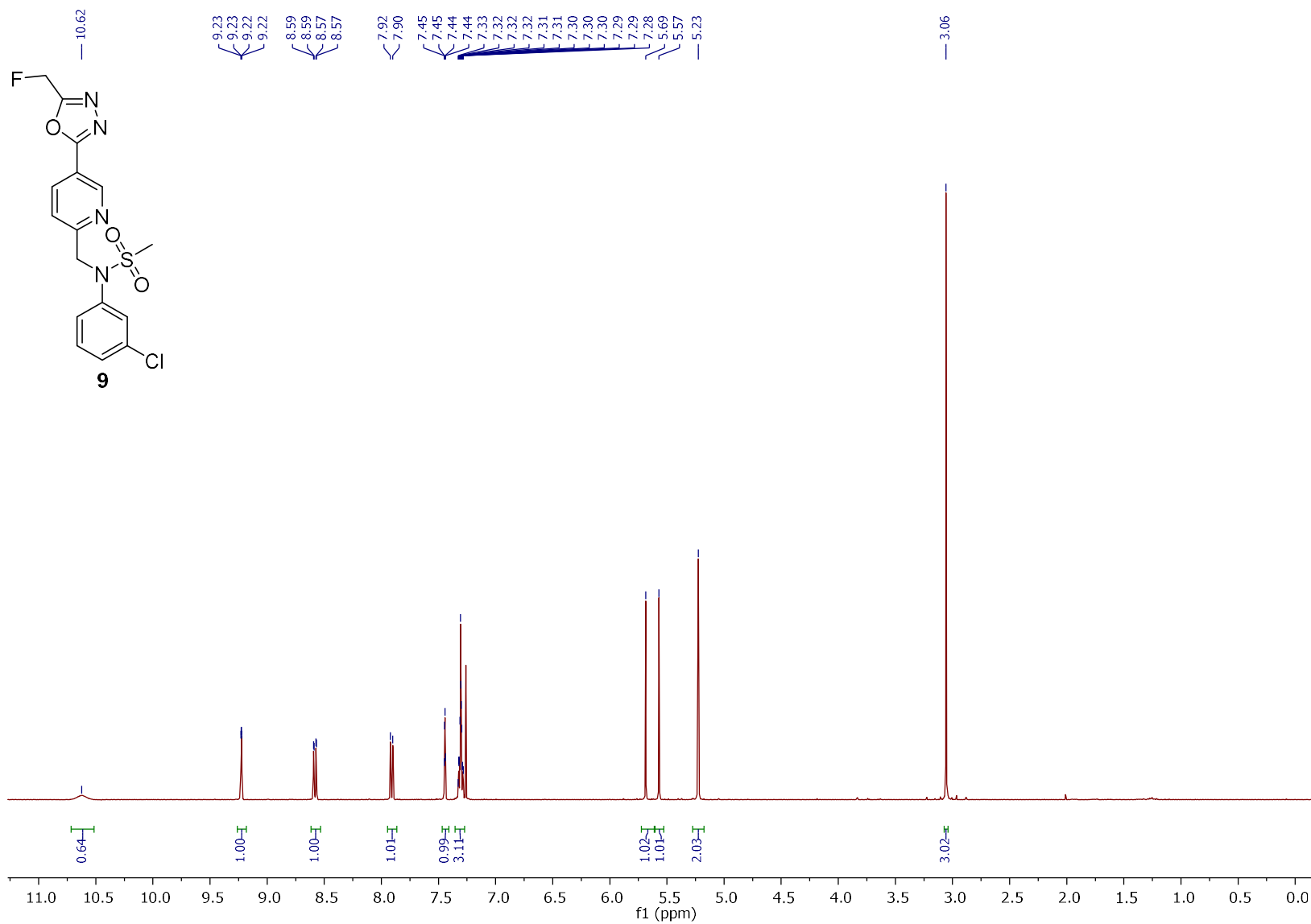

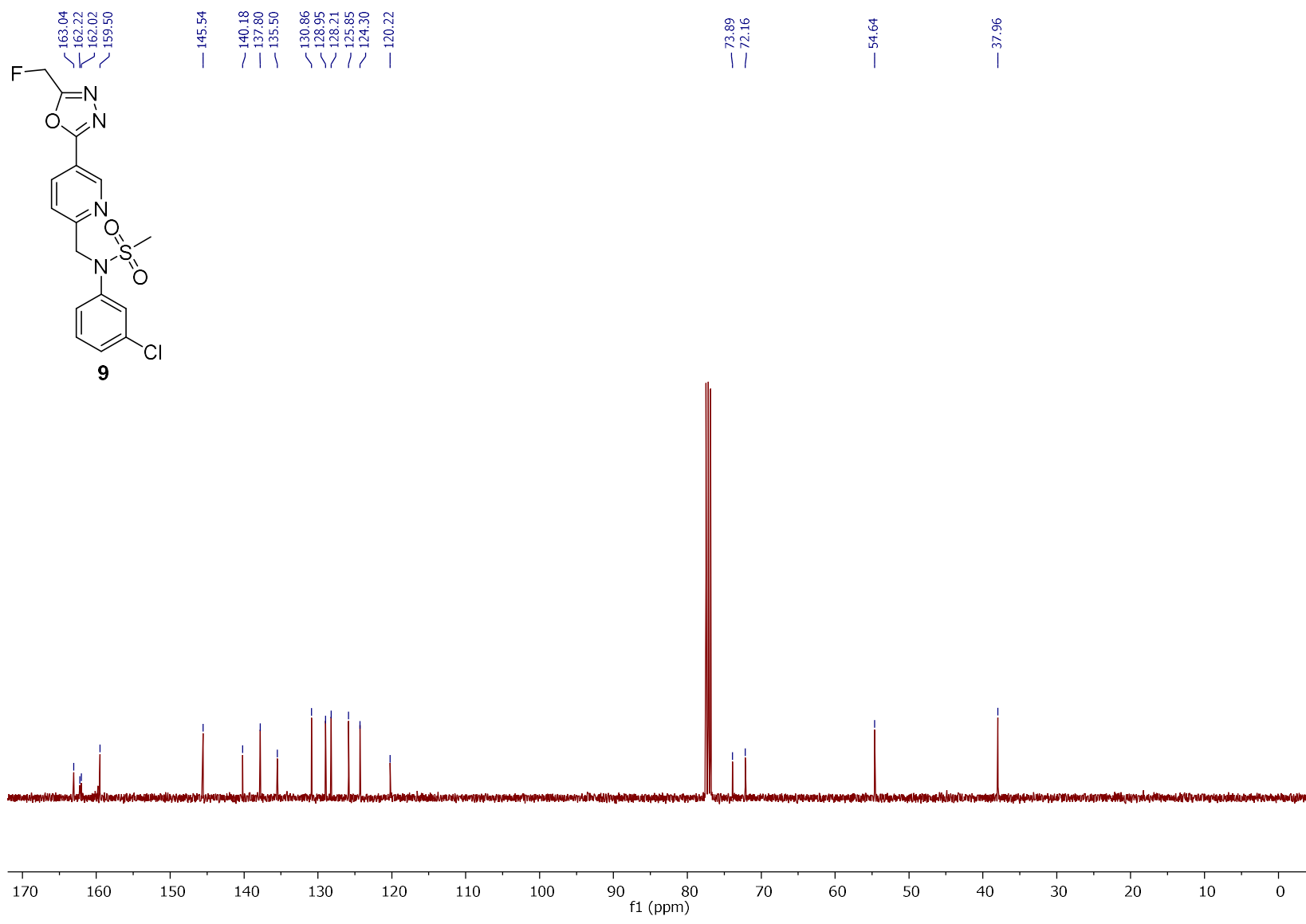

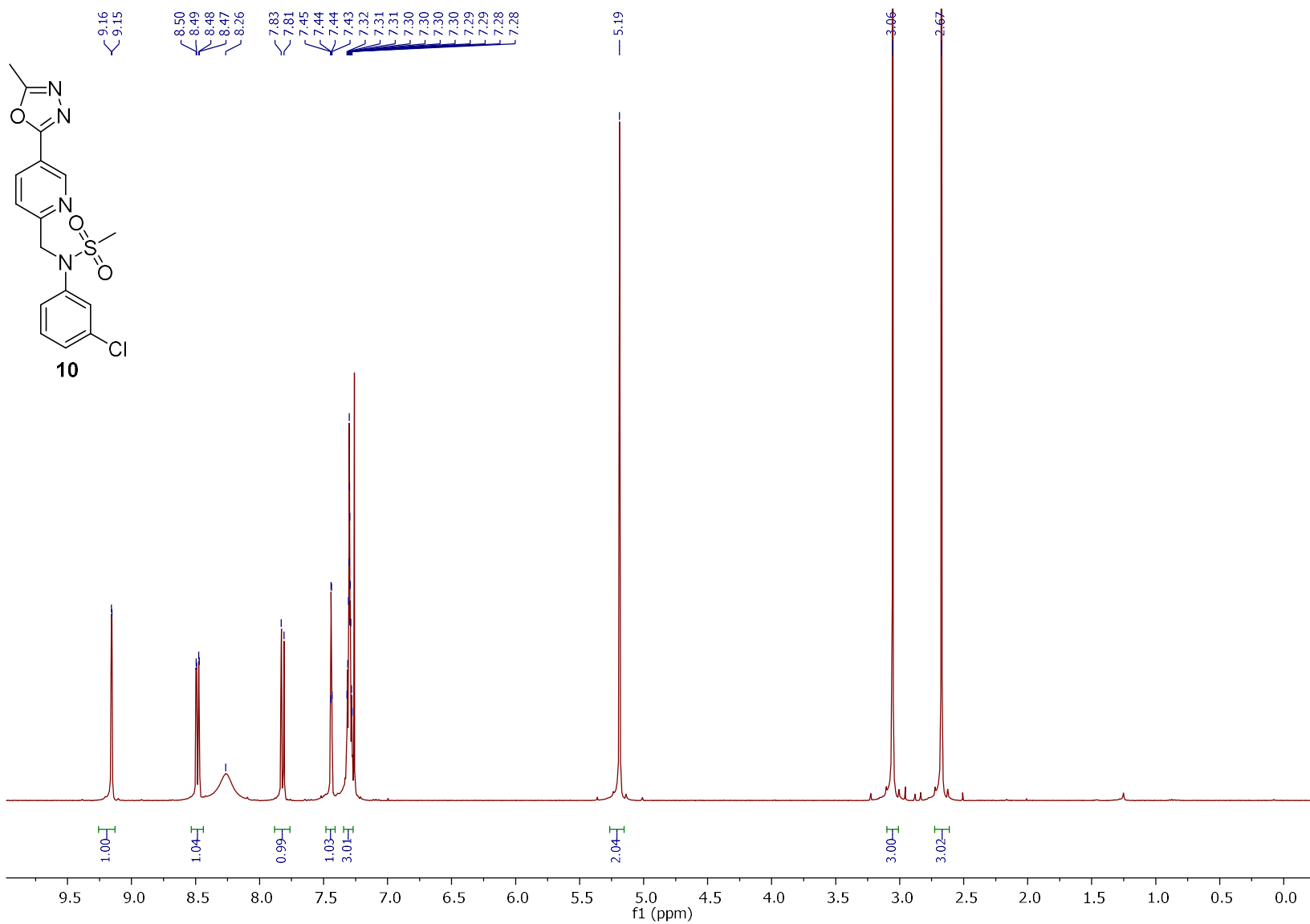

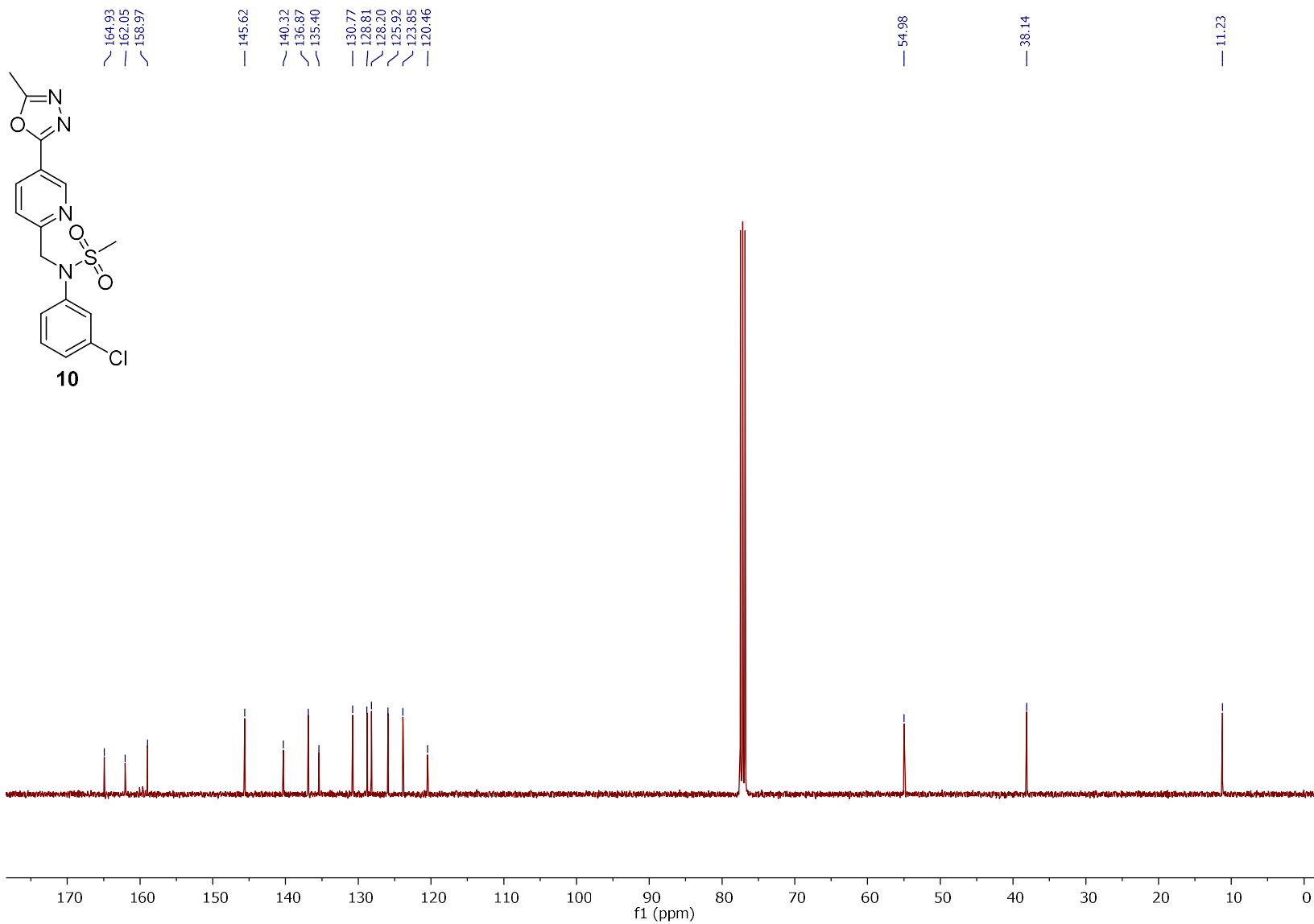

Supplement: Supplementary file 2 — cb3c00212_si_002.pdf [file cb3c00212_si_002.pdf]
